# Colonization of Mice With Amoxicillin-Associated *Klebsiella variicola* Drives Inflammation via Th1 Induction and Treg Inhibition

**DOI:** 10.3389/fmicb.2020.01256

**Published:** 2020-06-24

**Authors:** Huai Lin, Qing Wang, Lei Liu, Zeyou Chen, Ranjit Das, Yanhui Zhao, Daqing Mao, Yi Luo

**Affiliations:** ^1^Ministry of Education Key Laboratory of Pollution Processes and Environmental Criteria, College of Environmental Sciences and Engineering, Nankai University, Tianjin, China; ^2^Hebei Key Laboratory of Air Pollution Cause and Impact (preparatory), College of Energy and Environmental Engineering, Hebei University of Engineering, Handan, China; ^3^School of Medicine, Nankai University, Tianjin, China

**Keywords:** amoxicillin, colitis, *Klebsiella variicola*, proinflammation, Th1/Treg balance

## Abstract

β-Lactam antibiotics can increase the resistance and virulence of individual intestinal microorganisms, which may affect host physiology and health. *Klebsiella*, a crucial gut inhabitant, has been confirmed to be resistant to most β-lactam antibiotics and contributes to the etiology of inflammatory bowel disease (IBD). In this study, the influence of amoxicillin (AMO) on *Klebsiella* and its role in colitis was investigated in an antibiotic cocktail (ABx) murine model. The results suggested that a 7-day AMO treatment significantly enriched the abundance of *Klebsiella* and enhanced serum resistance, antibiotic resistance, and biofilm formation ability of *Klebsiella variicola* (*K. variicola*) compared to the wild-type strain in the control group mice. Colonization of mice with the AMO-associated *K. variicola* could induce Th1 cells and inhibit Treg differentiation to promote inflammation in ABx murine model. In addition, inoculation of AMO-associated *K. variicola* in dextran sodium sulfate (DSS)-induced colitis murine model mice also confirmed that *K. variicola* colonization exacerbated inflammation as assessed by increased TNF-α, IFN-γ, IL-17a, and disease activity (DAI) levels; decreased colon length and bodyweight; and a disrupted Th1/Treg balance. The results of our study demonstrate that AMO enhances *Klebsiella* virulence in mice by disrupting the T cell equilibrium to exacerbate colitis, thereby providing a reference for proper antibiotic prescription.

## Introduction

The gut microbiota has been recognized as being indispensable for maintaining host homeostasis via nutrient production and processing and immune system training (Samuli et al., 2012). Furthermore, the perturbation of the gut microbiota can promote infections, metabolic syndromes, and neurological and inflammation diseases (Menchén, 2009; Cristina et al., 2012; Rooks and Garrett, 2016; Sharon et al., 2016). Antibiotics indiscriminately kill or prevent the growth of both pathogenic and commensal bacteria, having a long-lasting impact on gut microbiota (Roberfroid et al., 2010; Maurice et al., 2013; Mikkelsen et al., 2016). Antibiotic exposure has been demonstrated to not only alter the composition and function of the gut microbiota and upregulate the expression of antibiotic resistance genes (ARGs) but also affect health by causing asthma, allergies, diarrhea, obesity, diabetes, and inflammatory bowel diseases (IBDs) in both early life and adulthood (Lewis and Pamer, 2017; Ni et al., 2017; Mccoy et al., 2018). While microbial disruption due to antibiotic has been linked to increased susceptibility to these diseases, a causal relationship has not yet been established. Amoxicillin (AMO), a β-lactam antibiotic, is one of the most widely used antibiotics worldwide due to its safety (Atli et al., 2016). Recent studies found that AMO disrupted the gut microbiota and induced ARGs that may increase the healthy risk (Korry et al., 2020; Wang et al., 2020). However, the impact of AMO on microbiome-associated diseases remains lacking. It was previously reported that penicillin-perturbed gut microbiota could be transferred to offspring, making them more susceptible to the development of colitis (Schulfer et al., 2018). In addition, ampicillin has been shown to affect the virulence and resistance of the gut microbiota by altering bacterial toxin production (Goneau et al., 2015). However, most studies have primarily focused on analyzing microbial composition and function disruption in response to antibiotic exposure (Cekanaviciute et al., 2017). An adequate understanding of the relationship between the effects of antibiotics toward specific bacteria and the paradigm of such alterations in associated disease pathogenesis remains lacking.

*Klebsiella* spp. are members of the intestinal flora and are among the most common intestinal opportunistic pathogens that are widely present in water, soil, and air, promoting their spread and causing infections among the immunocompromised population (Bengoechea and Sa Pessoa, 2019). These microbes can quickly acquire exogenetic genes through horizontal gene transfer from other environmental bacteria, and the resulting increase in virulence and antibiotic resistance increases their ability to cause infection in community and hospital settings (Tzouvelekis et al., 2012; Bengoechea and Sa Pessoa, 2019). Importantly, no less than one-third of *Klebsiella pneumoniae* (*K. pneumoniae*) isolates have been confirmed to be resistant to at least one antibiotic (Munoz-Price et al., 2013; Qin et al., 2014). In particular, β-lactam-resistant *K. pneumoniae* capable of carbapenemase production have spread across many countries and regions (Munoz-Price et al., 2013). Once exposed to these resistant pathogenic bacteria, incurable infections may occur (Patel et al., 2008). *In vitro* experiments have demonstrated that long-term low-dose antibiotic treatment can induce bacterial toxin production and biofilm formation and facilitate antibiotic resistance (Goneau et al., 2015). However, the effect of antibiotics on enhancing the antibiotic resistance and virulence of *Klebsiella* species *in vivo* remains unclear.

The intricate interactions between multidrug and hypervirulent *Klebsiella* species and the immune system may cause difficulties for therapeutic interventions (Bengoechea and Sa Pessoa, 2019). Germ-free animals bred in sterile environment was used for such comparative studies (Henrik et al., 2011). However, such a mouse model that displayed an immature and disordered immune system is expensive and requires specific infrastructure (Smith et al., 2007; Henrik et al., 2011). Generally accessible alternative broad-spectrum antibiotic administration against both Gram-positive (ampicillin and vancomycin) and Gram-negative (ampicillin and neomycin) aerobic, facultative strains and anaerobic (metronidazole), substantial deplete gut microbiota (antibiotic cocktail, ABx) in mice that possessed germ-like phenotype was therefore more used as an alternative model for germ-free animals to study host–microbe interaction (Rakoff-Nahoum et al., 2004; Henrik et al., 2011). It has been studied that Enterobacteriaceae are essential modulators of colitis severity (Sovran et al., 2018). *Klebsiella* can be recognized through receptors such as Toll-like receptors (TLRs) or nucleotide-binding and oligomerization domain-like receptors (NLRs). These multidrug and hypervirulent pathogenic bacteria further promote the differentiation of T cells and stimulate the production of cytokines, such as IL-1β, IL-18, TNF-α, IL-22, and IL-17, which promote inflammation (Happel et al., 2003; Takeuchi and Akira, 2010; Cai et al., 2012; Boxx and Cheng, 2016; Friedrich et al., 2019). Murine experiments have demonstrated that Th1 and Th17 cells play proinflammatory roles in many bacterial-induced intestinal diseases, while Tregs have been shown to have anti-inflammatory and tolerance-maintaining functions (Atarashi et al., 2017; Saleh et al., 2019). Recently, some studies have revealed that the ectopic colonization of oral *Klebsiella* strains in the intestine drives Th1 cell induction and inflammation through TLRs and IL-18 signaling of the dendritic cell-mediated pathway using germ-free mice (Atarashi et al., 2017). Although these studies have confirmed the role of *Klebsiella* in pathogenesis of inflammation, how the virulence and resistance of the pathogens affect the immune response during the pathogenesis of colitis remains poorly understood.

Therefore, in this study, it was hypothesized that antibiotic-dependent variations in specific bacteria *in vivo* would enhance the risk of colitis in mice. First, we investigated the influence of AMO on *Klebsiella* antibiotic resistance and virulence. Then, based on the results of these assays, an antibiotic cocktail (ABx; consisting of vancomycin, neomycin, colistin, and ampicillin) murine model was used to explore how the alterations in *Klebsiella* abundance and activity induce the host Th1/Treg immune reaction and exacerbate inflammation. In addition, the pathogenic role of immune system alterations caused by *Klebsiella* on a dextran sulfate sodium (DSS)-induced colitis murine model was assessed. These findings revealed that antibiotics enhanced *Klebsiella* virulence by altering Th1/Treg differentiation to exacerbate colitis.

## Materials and Methods

### Bacterial Strain Identification and Inoculum Preparation

The 6- to 8-week wild-type specific-pathogen-free mice (C57BL/6J) from Huafukang Company (Beijing, China) were acclimated to animal facility for 1 week and then randomly divided into two groups that administered a single course of AMO (AMO-treated group, *n* = 10) or water (control group, *n* = 10) three times per day for 7 days. Mice were housed in standard specific-pathogen-free colonies at the Laboratory Animal Resource Center of Institute of Radiation Medicine, Chinese Academy of Medical Sciences and Peking Union Medical College following relating ethical approvals of Animal Ethics and Welfare Committee (Project IRM-DWLL-2016121). During antibiotic exposure experiment, mice were maintained on a 12-h light/dark cycle with free access to food and water. AMO dosage of 25.0 mg⋅kg^–1^ gavaged three times for mice per day was based on the therapeutic dose achieved peak effective bacteriostatic serum concentration in human children equal to that achieved in a similar serum concentration in mice (Andes and Craig, 1998; Fonseca et al., 2013; Nobel et al., 2015; Atli et al., 2016)Craig. Three aliquots of fresh fecal samples were randomly collected from the mice in these two groups and immediately added to PBS at a ratio of 1:9 (feces/PBS; W/V). Subsequently, 100 μL of the mixture was transferred to MIAC medium to promote *Klebsiella* growth as previously described (Atarashi et al., 2017). A total of 28 strains were obtained, from four to five colonies of each fecal sample (Supplementary Tables S2, S3). Subsequently, the total of 28 strains were identified by 16S rRNA gene sequencing using the full-length 16S-amplicon primers 27F (5′-AGAGTTTGATCCTGGCTCAG2-3′) and 1492R (5′-GGTTACCTTGTTACGACTT-3′) (Frank et al., 2008). Because 18 colonies matching *Klebsiella* account for 64% of all identified strains (*K. variicola* occupied 78% of the identified *Klebsiella*, Supplementary Tables S2, S3), *K. variicola* was chosen for further analysis. Representative *K. variicola* strains K6 and Z5 were thus picked from the control and AMO-treated groups, respectively. Both *K. variicola* strains (K6 and Z5) were grown in Luria-Bertani (LB) medium overnight at 37°C. Subsequently, 200 μL of bacterial suspensions in water (approximately 5 × 10^9^ CFU mL^–1^) were administered to antibiotic cocktail (ABx) or dextran sodium sulfate (DSS)-treated mice by intragastric gavage.

### Bacterial 16S rRNA Gene Sequencing

DNA was extracted from fecal samples collected from the AMO-treated and control groups using a Magen HiPure Stool DNA kit according to the manufacturer’s instructions. The V3–V4 region of the bacterial 16S rRNA gene was amplified and sequenced using an Illumina MiSeq platform at Majorbio Bio-pharm Technology Co., Ltd. (Shanghai, China). The data analysis was performed on I-sanger platform of Majorbio^1^. The sequenced 16S rRNA data were deposited in NCBI Sequence Read Archive under the accession number SAMN12429552–SAMN12429557.

### Whole-Genome Sequencing

Genomic DNA was extracted from *K. variicola* strains Z5 and K6 using a QIAamp DNA kit (Qiagen). Whole-genome sequencing was performed using an Illumina HiSeq X10 PE150 platform. The average nucleotide identity (ANI) was calculated according to a previously established method (Goris et al., 2007)^2^. The sequence data were deposited at NCBI under the accession numbers JAAGKS000000000 and JAAGKT000000000.

### Antimicrobial Susceptibility Testing

The minimum inhibitory concentrations (MICs) of *K. variicola* K6 and Z5 for chloramphenicol (chl), ampicillin (amp), kanamycin (kan), gentamicin (gen), neomycin (neo), imipenem (ipm), streptomycin (str), and tetracycline (tet) were determined as previously described (Clinical and Laboratory Standards Institute [CLSI], 2009). Briefly, *K. variicola* cell suspensions were prepared using an overnight culture at a concentration of 0.5 MCF (*n* = 3 for each bacteria). Then, 100-μL aliquots of the suspensions were added to 96-well microplates containing 100 μL of serially diluted antibiotics (512 mg⋅L^–1^ to 1 mg⋅L^–1^) prepared in MH (B)medium. The optical density at 600 nm (OD_600_) of each well was determined using an ELISA plate reader after 20 h of incubation at 37°C. The MIC values were reported as the concentration of antibiotics that inhibited 95% of bacterial growth in broth.

### Serum Resistance Assays

Serum resistance assays were performed as previously described (Gu et al., 2018). Aliquots (25 μl) of bacterial suspensions (10^6^ CFU⋅mL^–1^ in saline) were mixed in 96-well microplates with 75 μl of fresh, unheated human serum collected from healthy adult volunteers (*n* = 3 for each test). The microplates were incubated for 3 h at 37°C, and 100-μL aliquots collected at 60, 120, and 180 min were plated onto LB agar-solidified medium. The plates were incubated overnight at 37°C, and viable bacterial colonies were enumerated. Inactivated serum preincubated at 65°C for 30 min was used as a negative control. The enumerated cells were used to calculate the survival rate.

### Biofilm Formation Assays

Biofilm assays were performed in 24-well plates following previously described methods with some modifications (Khalil et al., 2019). Briefly, polymers were cut into 0.5-cm pieces and then sterilized. Three overnight cultures of *K. variicola* as same dilutions (10^6^CFU⋅mL^–1^) were used to inoculate 24-well plates containing five polymer pieces. The biofilm biomass was quantified after 48 h of growth at 37°C using crystal violet staining as described elsewhere (Khalil et al., 2019). Assays were performed in quintuplet, and OD_570_ values were analyzed using GraphPad eight by *t* test to evaluate the differences in biofilm formation between the strains.

### Animal Treatment

All animal experiments were conducted in accordance with the guidelines for animal care and use of the Chinese Academy of Medical Sciences and Peking Union Medical College following related ethical approvals of the Animal Ethics and Welfare Committee (Project IRM-DWLL-2016121).

Six- to eight-week-old specific-pathogen-free male C57BL/6J mice (Huafukang Co., Ltd., Beijing, China) weighing 18–25 g were used in this study. The animals were allowed to acclimate for 1 week before the study began. All mice were bred in standard specific-pathogen-free colonies at the Institute of Radiation Medicine, Chinese Academy of Medical Sciences and Peking Union Medical College were allowed *ad libitum* access to sterile food and water and maintained on a 12-h light/dark cycle. Before treatment, all mice were randomly divided into six groups. The mice were then prepared for inoculation by the oral delivery of antibiotics (low aspartame-based sweetener added for good taste to mice) or water in the drinking water (1.0 g⋅L^–1^ neomycin, 1.0 g⋅L^–1^ ampicillin, 1.0 g⋅L^–1^ metronidazole, and 0.5 g⋅L^–1^ vancomycin) for 14 days. Such duration of 14 days antibiotic treatment was chosen based on bacteria absent (<1 CFU⋅mg^–1^ in 10^7^-fold dilutions) from feces aerobes (37°C for 48 h) and anaerobes (37°C for 48 h in an anaerobic chamber) incubation plated on blood agar medium. The antibiotic cocktail was freshly prepared every 36 h. After 14 days, all antibiotic-treated mice were randomly divided into five groups: the Antibiotic group (administrated water, *n* = 10); the K6 group (administered the *K. variicola* strain K6 isolated from the feces of the control mice, *n* = 10); the Z5 group (administered the *K. variicola* Z5 strain isolated from the feces of the AMO-treated mice, *n* = 10); the K6D group (7 days DSS treatment and then administered the *K. variicola* strain K6 isolated from the feces of the control mice, *n* = 10); and the Z5D group (7 days DSS treatment and then administered the *K. variicola* strain Z5 isolated from the feces of the AMO-treated mice, *n* = 10). The K6D and Z5D groups were given 2.5% (wt/vol) DSS (Meilun Biotechnology Co., Ltd, Dalian, China) in drinking water *ad libitum* for an additional 7 days after the antibiotic treatment. In addition, the K6 and Z5 groups were administered the K6 and Z5 strains at 10^8^ CFU for 7 days. After the DSS treatment, the K6D and Z5D groups were inoculated with K6 and Z5 for the following 7 days. The water-treated mice were used as a healthy control group without antibiotic treatment (received water throughout the experiment, *n* = 10). The control and antibiotic group mice were euthanized along with K6 and Z5 at 21 days or K6D and Z5D treatment at 27 days. The body weight of the mice was monitored daily, while colon length was measured at the end of the experiment. Disease activity was scored as previously described (Zhang et al., 2016).

### Histology and Cytokine Quantification

About 1-cm proximal colon sections located between ascending and transverse colon (1.5 cm from cecum) were harvested. Colon pieces were fixed in 10% neutral-buffered formalin, embedded in paraffin, and then stained with hematoxylin-eosin (H&E) for histological analysis. Histological analysis was performed in a blinded manner as previously described (Goneau et al., 2015). Serum samples were collected and stored at −80°C until further processing, and ELISA was performed using the serum samples according to the manufacturer’s instructions (ABclonal Inc., Wuhan, China).

### Mouse Immune Cell Isolation and Flow Cytometry

Mesenteric lymph nodes, cervical lymph nodes, blood, and spleens were collected from the mice, and the samples were cut into small pieces and forced through a 100-μm cell strainer. Single-cell suspensions were stimulated with 40.5 ng⋅mL^–1^ of phorbol-12-myristate-13-acetate (PMA) and 700 ng⋅mL^–1^ of ionomycin in the presence of 2.5 μg⋅mL^–1^ of a protein transport inhibitor (Biolegend, #423303) for 6–8 h at 37°C. Then, the cells were incubated in 5% of fetal calf serum for 15 min and stained with anti-CD45-PerCP (Biolegend, #103130), anti-CD4-FITC (Biolegend, #100510), and anti-IFN-γ-PE/Cy7 (Biolegend, #505825). Aliquots of the cells were fixed and permeabilized with FoxP3/True-Nuclear^TM^ Transcription factor buffer set (Biolegend, #424401) and stained with anti-FoxP3-PE (Biolegend, #320007), anti-CD45-PerCP (Biolegend, #103130), and anti-CD4-FITC (Biolegend, #100510).

### Statistics

Average values and standard deviations were prepared and calculated in GraphPad Prism version eight (San Diego, CA, United States). For all data displayed in graphs, the results are expressed as the mean ± SD (*n* = 3–7 mice for each group). Student’s *t*-test was used for two-group comparisons and one-way analysis of variance (ANOVA) followed by Turkey’s *post hoc* test was used for more than two groups. Differences with a *P*-value less than 0.05 were considered statistically significant.

## Results

### The Antibiotic Resistance and Virulence of *K. variicola* Are Enhanced in AMO-Treated Mice

**FIGURE 1:**
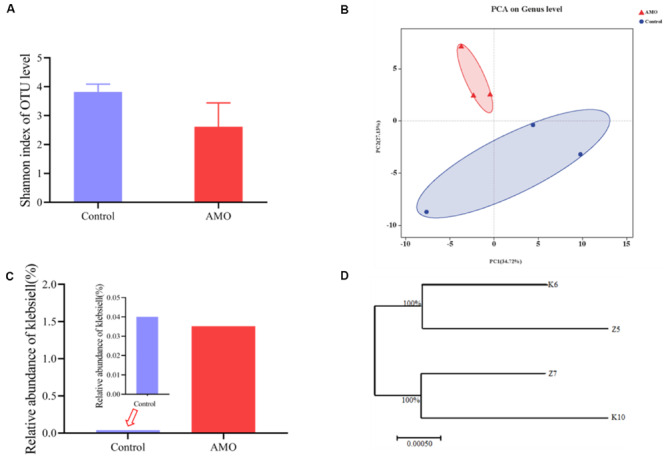
Effect of AMO on the mouse gut microbial community. **(A)** Diversity of microbiota after AMO treatment, assessed by Shannon index. **(B)** PCA plot based on the genus level between the AMO and control groups. **(C)** Relative percentage of *Klebsiella* between the AMO and control groups. **(D)** Cladogram of 4 representative *Klebsiella* isolated from AMO and control based on the full-length 16S rRNA gene sequencing results. Data presented as means ± SD (*n* = 3), difference at significance level *P < 0.05 (*t*-test) between two groups.

The 16S rRNA gene was PCR amplified from mouse feces samples and sequenced to assess the influence of AMO on the microbiota. In this study, the mice treated for 7 days with AMO exhibited significantly reduced microbial community diversity compared to that observed in the control group (*P* < 0.05, Figure 1A). In agreement with these results, the structure of the gut microbiota was also disrupted. The principal component analysis (PCA) results showed that the microbial taxa in the AMO treatment group clustered separately from that of the control group (Figure 1B). More importantly, the relative abundance of *Klebsiella* (*Proteobacteria*) was increased by 41-fold in the AMO treatment group compared with the control group (Figure 1C). In addition, the abundance of human diseases categories (KEGG level 1) was significantly increased in the AMO group (Supplementary Figure S1A, *P* < 0.05). Further correlation analysis found that *Klebsiella* significantly correlated with enriched abundance of bacterial invasion of epithelial cells (KEGG level 3 of human diseases) (Supplementary Figure S1B, *R* > 0.9, *P* < 0.01). Because *Klebsiella* species has been proved to interfere with host inflammation and increases in their abundance can cause human diseases (Bengoechea and Sa Pessoa, 2019), this microbe was selected for further study.

Culture-based methods and whole-genome sequencing were used to identify the isolated strains. Bacterial colonies grown on MacConkey inositol adonitol carbenicillin agar (MIAC) cultured from both the AMO treatment and control groups showed that OTUs mapped to *Klebsiella* were confirmed to be *K. variicola*. Two representative strains, designated as *K. variicola* K6 (isolated from the control group) and *K. variicola* Z5 (obtained from the AMO-treated group), were chosen based on the following criteria (Cekanaviciute et al., 2017): (1) they were present in both the control and AMO treatment groups to distinguish the antibiotic effect, (2) they have been reported to alter the immune response, (3) they are widely distributed pathogens associated with high morbidity and mortality, and (4) they were culturable to ensure their reproducibility. The multilocus sequence typing (MLST) analysis results using whole-genome sequencing data showed that two selected strains belonged to ST197. The ANI based on the genome sequencing data showed that the two strains have very high genomic similarity to each other (approximately 100%), suggesting that the two strains belong to the same species (Figure 1D, Supplementary Figure S2). These results indicated that *K. variicola* Z5 was the AMO-influenced strain corresponding to the wild-type strain K6, qualifying them for further study.

**FIGURE 2:**
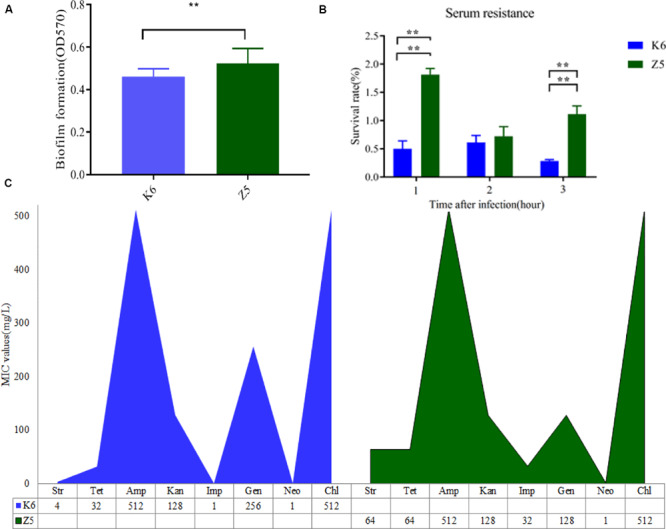
Virulence potential and antibiotic resistance profiles of *K. variicola* Z5 and K6. **(A)** Biofilm formation quantified by crystal violet staining and analysis at OD_570_ (*n* = 3). **(B)** Human serum resistance assays were performed using healthy human serum to assess the survival rate of 1 × 10^6^ colony-forming units of each strain after 3 h (*n* = 3). **(C)** Antibiotic resistance characteristics of all strains using CLSI antimicrobial susceptibility testing standards (Clinical and Laboratory Standards Institute [CLSI], 2009). The different colors represent different strains, with the height of the column indicating the MIC value of the corresponding antibiotic (*n* = 3). Data presented as means ± SD (*n* = 3), difference at significance level *P < 0.05, **P < 0.01 (*t*-test) between two groups.

Both the antibiotic resistance and virulence of *K. variicola* significantly increased with the AMO treatment in the mouse intestine (Figures 2A,B). Antimicrobial susceptibility assay results showed that the MICs of imipenem and streptomycin were notably increased to 32 and 64 mg⋅L^–1^ in *K. variicola* Z5 compared to only 1 and 4 mg⋅L^–1^ in *K. variicola* K6, respectively (Figure 2C). Furthermore, the biofilm formation and serum resistance abilities of *K. variicola* Z5 were also significantly greater than those observed for *K. variicola* K6 (*P* < 0.01, Figures 2A,B).

### Colonization of Mice With AMO-Associated *K. variicola* Induces Inflammation

**FIGURE 3:**
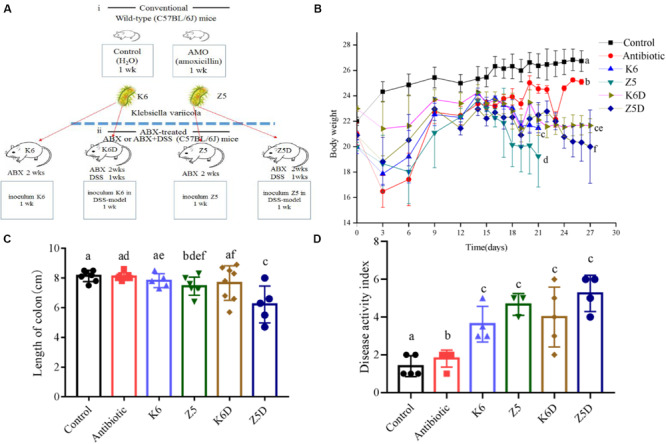
Increased proinflammation in colonized AMO-resistant *K. variicola* in ABx-treated or DSS-induced colitis mice. **(A)** Experimental design for colonization with each *K. variicola* strain in the ABx model. **(B)** Weight loss was reported as the mean ± SD from each group. **(C)** The length of the colon measured during treatment. **(D)** Disease activity index. Different letters indicate significant differences (*P* < 0.05, one-way ANOVA) between different groups (mean ± SD, *n* = 3–8 mice per group).

**FIGURE 4:**
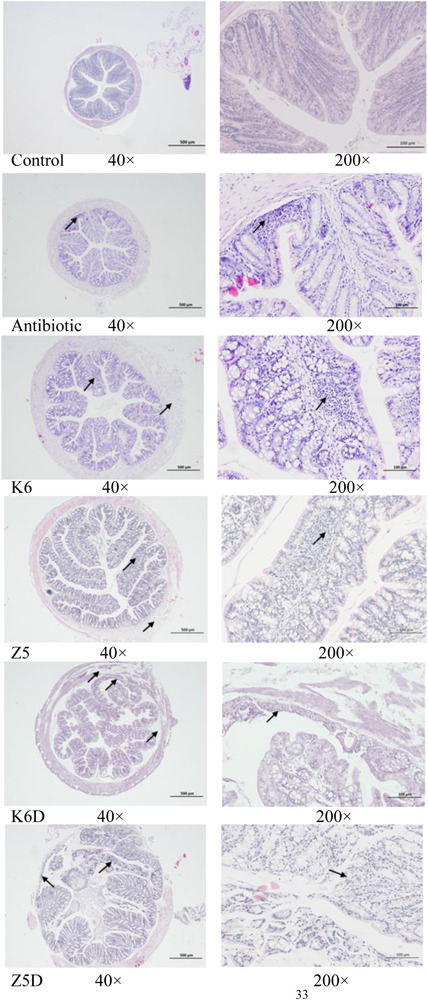
Histopathological analysis of the effects of AMO-resistant *K. variicola* on mouse colons in the ABx-treated or DSS-induced colitis mouse model. Representative H&E staining of the proximal colon 7 days after treatment. The arrow indicates lesions. The scale bar is labeled in the image.

ABx-treated mice were inoculated with AMO-associated *K. variicola* Z5 and K6 strains to assess the effects of these species on inflammation during the AMO treatment of mice (Figure 3A). Control group treated with water throughout the experiment was set to healthy control in that its body weight shows normal growth. Compared to the healthy control, mice treated with multiple antibiotics lost a little weight with no detectable distress or pain within 14 days, which allowed us to further compare the specific organism’s effect (Figures 3B,C, *P* > 0.05). Consistent with the enhanced serum resistance and biofilm formation, *K. variicola* Z5 administration induced an increase in inflammation activity in ABx-treated mice that were characterized by significant decreases in weight, shorter colon lengths, and increase in higher disease activity index (DAI) values compared with those observed in the *K. variicola* K6, control, and antibiotic groups (*P* < 0.05, Figures 3B–D). Specifically, *K. variicola* Z5 significantly increased the serum levels of the proinflammatory cytokine IL-17a and mildly increased those of the inflammation markers TNF-α and IFN-γ (*P* > 0.05) (Supplementary Figure S3). In addition, the levels of the anti-inflammatory cytokine IL-10 were also increased in the *K. variicola* Z5 group (Supplementary Figure S3). Mice administered *K. variicola* Z5 exhibited more severe colonic damage that was revealed by occurrence of loosely arranged muscle fibers in muscle layers and inflammatory cell infiltration in mucous layer compared with that observed in the control group based on the results of histological structure analyses (Figure 4). In contrast, the mice administered *K. variicola* K6 exhibited almost no inflammation as assessed by observations of an insignificant variation in colon length, DAI value, serum IL-10, TNF-α, and IFN-γ levels and histological damage compared with that observed in the control mice (*P* > 0.05, Figures 3B–D, Supplementary Figure S3). Thus, these results indicated that *K. variicola* Z5 was responsible for the observed alterations in inflammation cytokine levels and colon histological structure.

### AMO-Associated *K. variicola* Induces Th1 Cells and Inhibits Treg Differentiation

**FIGURE 5:**
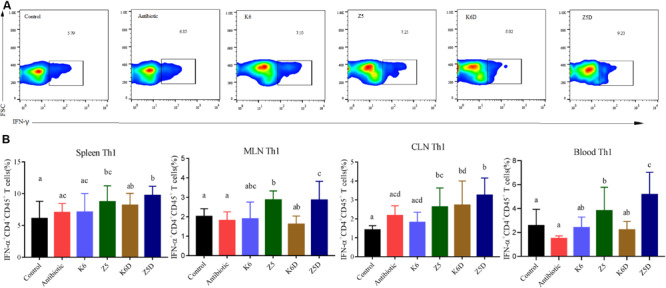
Th1 cell induction and its role in DSS-induced colitis in mice model. **(A)** Data represent flow plots and quantification of Th1 cell portion of spleen. **(B)** In the spleen, MLN, CLN, and blood in each group. Different letters indicate significant differences (*P* < 0.05, one-way ANOVA) between different groups (mean ± SD, *n* = 3–7 mice per group).

**FIGURE 6:**
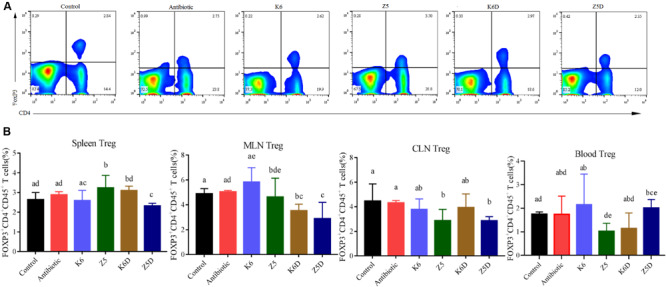
Treg inhibition and its role in DSS-induced colitis in mice. **(A,B)** Flow plots and quantification of Tregs in the spleen and in the spleens, MLNs, CLNs, and blood in each group. Different letters indicate significant differences (*P* < 0.05, one-way ANOVA) between different groups (mean ± SD, *n* = 3–7 mice per group).

AMO associated *K. variicola* Z5 induced inflammation by modulating Th1/Treg differentiation. In the ABx-treated mice colonized with *K. variicola* Z5, T cell differentiation was assessed in multiple peripheral lymphoid tissues, including the spleens, blood, mesenteric lymph nodes (MLNs) and cervical lymph nodes (CLNs). Upon evaluation of the antibiotic and control group at 21 and 27 days, we found that multiple antibiotics did not significantly change the Th1 or Treg after antibiotic cessation (Supplementary Figure S4, *P* > 0.05). That may mean that neither 14 days of multiple antibiotic treatment nor 7 days (from 14 days ABx) or 14 days (from 21 days ABx) of microbial re-established after antibiotics within the gut were sufficient to have an impact on Th1 or Treg. We deem the 21- and 27-day samples of control and antibiotic as two combined whole groups in the following discussion. The results showed that *K. variicola* Z5 significantly increased the proportion of Th1 cells in the spleens, blood, MLNs, and CLNs compared to that observed in the control group (*P* < 0.05), potentially exacerbating inflammation (Figures 5A,B). No apparent induction of Th1 cells was observed in the *K. variicola* K6 group (*P* > 0.05, Figure 5B). Treg differentiation in the spleen, MLNs, CLNs, and blood were also detected (Figures 6A,B). The results demonstrated that colonization of mice with AMO-associated *K. variicola* Z5 inhibited Treg differentiation in the CLNs and MLNs compared to that observed in the control group (*P* < 0.05, Figure 6B). In contrast, the proportions of Tregs from the spleens, CLNs, MLNs, and blood of mice in the *K. variicola* K6 group were almost equal to those observed in the control mice (*P* > 0.05, Figure 6B).

### AMO-Associated *K. variicola* Exacerbates Disease Severity in DSS-Induced Colitis

Colonization of mice with *K. variicola* Z5 recapitulated Th1/Treg differentiation and exacerbated DSS-induced colitis severity. Remarkably, the disease symptom DAI value was significantly increased, while the body weights and colon lengths of mice were notably decreased in the Z5D group (DSS model monocolonized *with* AMO-resistant *K. variicola* Z5) compared with that observed in the K6D group (DSS model monocolonized *with* wild *K. variicola* K6) or control group (*P* < 0.05, Figures 3B–D). Furthermore, the histological results showed enhanced colitis phenotypes with a loss of histological structure, necrosis of some intestinal gland cells, abscission of epithelial cell, and marked inflammatory cell infiltration in the mucous layers of mice in the Z5D group compared with the moderately injured K6D or control mice (Figure 4). In addition, the DSS-associated colitis immune alterations were also more obvious in the Z5D group compared with the K6D or control groups, as shown by increased serum levels of proinflammation cytokines, such as TNF-α, IFN-γ, and IL-17a (*P* < 0.05, Supplementary Figure S3).

The induction of Th1 cells and the inhibition of Tregs was recapitulated in a DSS-colitis model. The Z5D treatment group mice exhibited significantly increased Th1 cell induction in the spleens, blood, MLNs, and CLNs compared to that observed in mice from the K6D and control groups (*P* < 0.05, Figure 5B). Furthermore, decreased Treg proportion was also observed in the spleens, blood, MLNs, and CLNs (*P* < 0.05, Figure 6B), suggesting the inhibitory effect of AMO-associated *K. variicola* on lymphocyte differentiation in the DSS model (Figure 6B). The lack of Th1 induction and Treg inhibition was observed in both the K6D and control groups, which was consistent with the unaltered colitis symptoms (Figures 5, 6).

## Discussion

Recently, numerous studies have reported that antibiotics reduce the taxonomic richness, diversity, and evenness of the intestinal microbial community, which may increase the risk of disease development, such as IBD, asthma, and obesity, both in the host and even in their offspring (Khan et al., 2016; Mikkelsen et al., 2016; MacPherson et al., 2018; Schulfer et al., 2018). However, it is also important to explore how antibiotics influence specific microbes to affect host functions. *Klebsiella* has been shown to disrupt the immune system and induce inflammation, resulting in an increased susceptibility to IBD (Bengoechea and Sa Pessoa, 2019). Nonetheless, studies on the influence of initial antibiotic use on *Klebsiella* within the gut are rare. Our results showed that AMO disruption of the gut microbiota was accompanied by an enhancement of the virulence and antibiotic resistance of the opportunistic pathogen *K. variicola*. These results also showed that AMO associated *K. variicola* can exacerbate the DSS-induced colitis by inducing Th1 cells and inhibiting Treg differentiation in the mice.

### AMO-Associated Alterations in Intestinal *Klebsiella* Within the Gut

Identifying the mechanism, especially identifying the response of individual species, during antibiotic disruption is crucial for the development of precise therapies or microbial-associated dysbiosis preventative methods. Consistent with previous investigations of antibiotics, AMO treatment significantly reduced the diversity and perturbed the structure of the gut microbiota (Nobel et al., 2015; Schubert et al., 2015). Notably, in our study, the results of a genus-level analysis revealed that these responses were primarily attributed to a sharp increase in *Klebsiella* abundance. Reduced microbial diversity and a perturbed gut microbiota have been confirmed to be associated with immune disorder by several studies (Manichanh et al., 2006; Schulfer et al., 2018). Since AMO treatment induces microbiota disruption and is subsequently susceptible to diseases including colitis and gastrointestinal pathogen infection (Roubaud-Baudron et al., 2019), we would study the critical role of specific species. Nonetheless, due to limitations in 16S rRNA sequencing, alterations in specific bacteria among perturbed microbial communities cannot be evaluated at the species level and precisely identified within complicated bacterial community networks. The use of culture-based bacterial isolation methods enabled us to dissect the causal relationship between the microbial community and host well-being. Intriguingly, we found that the increased abundance of *Klebsiella* positively matches the bacterial invasion of epithelial cells. Furthermore, the observed enrichment of bacteria in the genus *Klebsiella* has been proved to affect the host’s immune system, and infections like Crohn’s disease and urinary tract infections (Atarashi et al., 2017; Cekanaviciute et al., 2017; Ni et al., 2017; Schirmer et al., 2019). Thus, in this study, *Klebsiella* strains were isolated from the AMO treatment and control groups as potential candidates to study the link between gut microbiota and colitis. Antimicrobial susceptibility, biofilm formation, and serum resistance test results revealed the enhanced antibiotic resistance and virulence of the AMO treatment group strain Z5, characteristics that are responsible for the hypervirulent potential of pathogens (Holt et al., 2015; Lam et al., 2018; Uzoechi and Abu-Lail, 2019). Although studies have shown that subinhibitory concentrations of antibiotics increase the biofilm formation of *E. coli in vitro* (Goneau et al., 2015), the rarely reported effects of AMO on enhancing the biofilm production and antibiotic resistance of the gut inhabitant *Klebsiella in vivo* were observed in our study. These findings suggest that alterations in *K. variicola* due to AMO treatment may promote inflammation.

### AMO Treatment-Associated Alterations in Strain Z5 That Promote Mild Inflammation May Be Self-Regulated

The association of biofilm formation with the virulence of pathogens has been confirmed to be correlated with inflammation, including a decrease in E-cadherin levels, an increase in IL-6 levels and activation of the STAT3 pathway (Tomkovich et al., 2019). Increased biofilm formation by bacteria is associated with increased inflammation (Goneau et al., 2015). Combined with the observed enhancement in virulence (as assessed by biofilm formation and serum resistance tests) of AMO-associated *Klebsiella*, our study also showed that *K. variicola* Z5 could induce inflammation. The strain Z5-colonized mice also exhibited enhanced colitis in a DSS-induced colitis model, whereas an inflammation phenotype was observed in the *K. variicola* K6-colonized or control mice. Thus, these results demonstrate that AMO-induced alterations in *K. variicola* may contribute to its immune evasion and an increased risk of colitis *in vivo*.

Cytokines, including IL-10, IL-6, IL-8, IL-1β, TNF-α, and IFN-γ, are produced in response to host–microbe interactions to defend the host and may be important for IBD development (Friedrich et al., 2019). The IL-10 cytokine family has been shown to facilitate the gut antimicrobial defense and promote mucosal barrier integrity and repair by activating STAT3 (Shouval et al., 2014), while IL-17, TNF-α, and IFN-γ induce colitis by disrupting the mucosal barrier function of the immune system (Leppkes et al., 2009; Friedrich et al., 2019). Studies have shown that the β-lactam antibiotic oxacillin stimulates the expression of lipoprotein-like (lpl) genes in methicillin-resistant *Staphylococcus aureus* (MRSA), which promotes the production of the proinflammation cytokines IL-6 and TNF-α in macrophages to trigger a TLR2-dependent immune response in mice (Shang et al., 2018). In agreement with these results, we showed that AMO treatment-associated changes in *K. variicola* also induce the production of proinflammatory cytokines such as cytokine IL-17a, TNF-α, and IFN-γ. Unlike previous studies, the levels of the anti-inflammatory cytokine IL-10 in mice treated with AMO-resistant *K. variicola* were also increased. This anti-inflammatory cytokine is important in suppressing IFN-γ and TNF-α by limiting macrophage activity, the production of which in mice was shown to be promoted by *Bacteroides fragilis* to inhibit colitis through the suppression of IL-17a production (Mazmanian et al., 2008). Such an increase in IL-10 may be due to antigen-specific Th1 cells, which have been shown to promote IL-10 production mediated by GPR43 (Sun et al., 2018). T-effector cell production of IL-10 was shown to be a self-regulated mechanism to prevent Th1 cell-induced inflammation (Sun et al., 2018), indicating that inflammation caused by AMO-resistant *K. variicola* may occur via the disruption of IL-10 levels. However, once *K. variicola* infection occurs, especially early on, the increased antibiotic resistance and virulence of this microbe caused by AMO administration may result in it being more difficult to treat. A recent study demonstrated that the multiple sequence types of extended-spectrum β-lactamases *K. variicola* isolates in intensive care unit present a large challenge for therapy (Giuffrè et al., 2016). Therefore, further attention needs to be paid to *Klebsiella* with respect to clinical AMO prescription.

### AMO-Associated *Klebsiella* Exacerbates Colitis by Regulating the Th1/Treg Balance

CD4 T cells play an essential role in the host immune system, regulating immune responses against a wide variety of pathogens via the differentiation of naïve CD4 T cells into Th1/Th2/Th17 cells and Tregs (Knochelmann et al., 2018). The balance of T cells, especially Th1/Th17 cells and Tregs, has emerged as having a prominent role in IBD (Knochelmann et al., 2018). Previous investigations revealed that antibiotics can significantly promote the development of colitis in IL10^–/–^ mice by perturbing the gut microbiota, indicating that antibiotics reshape the intestinal microbiota and can affect host physiology and health (Schulfer et al., 2018). Combined with the enhanced virulence of *K. variicola* observed in our study, we observed that AMO-resistant *K. variicola* promotion of Th1 cell induction and Treg inhibition triggers inflammation. These results are in agreement with those of a study by Atarashi, who showed that ectopic *Klebsiella* colonization of the gut of IBD patient induces an increase in the proportion of Th1 cells (Atarashi et al., 2017). In contrast, the opposite results were observed for *K. variicola* K6, which proved the effect of AMO on bacterial virulence in altering the Th1/Treg equilibrium. These results all demonstrate that antibiotic treatment altering individual bacterial species can disrupt the host T cell balance. However, in contrast to the immunomodulatory effect of AMO-associated *K. variicola*, whether the susceptibility of the host to variations in T cells play a crucial role in colitis remains to be determined. In this study, we showed that the AMO-associated strain Z5 exacerbated colitis in a DSS-induced mouse model. Human and mouse studies have proved that the host immune response, including with respect to T cell participation, influences the severity and outcome of colitis (Feghaly et al., 2013; Saleh et al., 2019). Notably, a recent study showed that the effect of colitis-associated inflammation on CD4 T cells affects the severity and mortality of *Clostridium difficile* infections (Saleh et al., 2019). Therefore, the ability of AMO-resistant *Klebsiella* to perturb the Th1/Treg balance may explain the pathogenesis of *K. variicola* in proinflammation and colitis. Once *Klebsiell*a gains access to the host, it is recognized by immune cells through pattern recognition receptors (PRRs) to induce an immune response. TLR4, TLR2, and TLR9 signaling have been shown to play an important role in *Klebsiella* infection recognition and defense. Mice deficient in TLR9 were failed to produce Th1 cells to clear bacteria (Urvashi et al., 2007; Ivin et al., 2017). Furthermore, TLR^–/–^ mice showed increased mortality when inoculated with *Klebsiella* due to an increase in the bacterial population in the lungs (Urvashi et al., 2007). In addition, TLR4^–/–^ × TLR2^–/–^ mice were also observed to be more susceptible to *Klebsiella* infection (Bengoechea and Sa Pessoa, 2019). However, the mechanism by which *Klebsiella* interacts with the immune system requires further study.

### Perspectives

Our study has limitations. While we demonstrate that AMO increased *Klebsiella* correlation with bacterial invasion of epithelial cells that verified to induce Th1 and inhibit Treg causing inflammation, the causality needs more vigorous discussion. First, more samples were not taken, including the association between microbiota and bacterial function, the comparative examination of colonic pathology, and the effect of *K. variicola* on inflammation due to costs and operational errors. Thus, the statistical analysis in this study only gives a glimpse of the virulence between control and AMO-associated *K. variicola* that warrants more sample size. Second, the antibiotic cocktail mouse model did not involve sterile mice such that we cannot exclude the possibility of recovery and expanded inhibition of commensal bacteria, especially the effect of other Gram-negative bacteria on T cell differentiation and inflammation. For example, *Acinetobacter* increased Th1, thus exacerbating inflammation (Cekanaviciute et al., 2017). Spatiotemporal *K. variicola* and its influence throughout the intestine was not established. Germ-free monocolonized mice of *K. variicola* were urgently needed to compare the colonization difference between small intestine and colon, especially in the mucosal layer to quantify the inflammatory response. Lastly, although we sequenced K6 and Z5, in-depth data mining was not well-performed. Genetic level of variation may well be explained by the virulence of the strains. Molecular biological validation to systemic *in vivo* and *in vitro* studies is required to definitively establish causal relationships between AMO and its perturbed *K. variicola* on host inflammation.

## Conclusion

Antibiotic exposure causes individual bacterial species (*K. variicola*) to have enhanced resistance and virulence. *K. variicola* was shown to induce Th1 cells and inhibit Treg differentiation, which triggered proinflammation and induced mild disruption of the colon histological structure. Furthermore, these alterations aggravated the development of DSS-induced colitis, indicating their role in the pathogenesis of colitis. These results demonstrate that increased attention needs to be paid to the variation in the virulence of individual pathogens in response to antibiotic treatment and its role in promoting diseases, supporting the need for appropriate antibiotic use to limit clinical *Klebsiella-*associated inflammation.

## Data Availability Statement

This Whole Genome Shotgun project has been deposited at NCBI SRA SRR9891530–SRR9891532 and SRR9891535–SRR9891537.

## Ethics Statement

The animal study was reviewed and approved by Chinese Academy of Medical Sciences and Peking Union Medical College.

## Author Contributions

YL, DM, QW, and HL conceived and coordinated the study. HL carried out the experiment, analyzed the data, and drafted the manuscript. LL and YZ carried out the laboratory work. ZC, LL, RD, and QW revised the manuscript. YL, DM, ZC, and QW provided the financial support. All authors read and approved the final manuscript.

## Conflict of Interest

The authors declare that the research was conducted in the absence of any commercial or financial relationships that could be construed as a potential conflict of interest.

## Footnotes

1 http://www.i-sanger.com

2 http://enve-omics.ce.gatech.edu/ani/index
