# Supplementary material for: Colonization of Mice With Amoxicillin-Associated Klebsiella variicola Drives Inflammation via Th1 Induction and Treg Inhibition
Source: Front Microbiol. 2020 Jun 24;11:1256. doi: 10.3389/fmicb.2020.01256 (PMC7326774; doi:10.3389/fmicb.2020.01256)
Supplement: Supplementary file 1 [file Data_Sheet_1.pdf]

SUPPLEMENT FIGURES

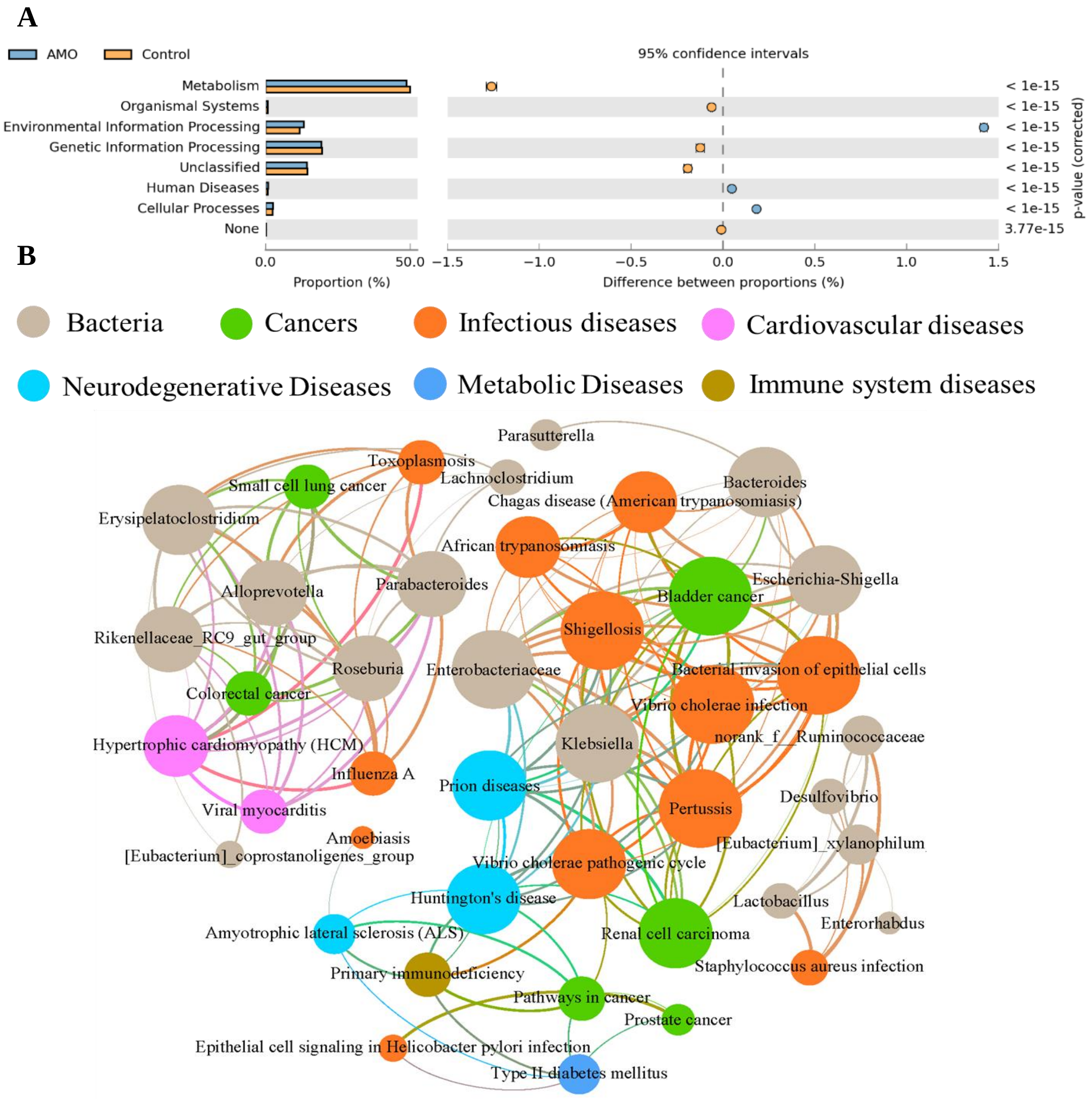

FIGURE S1 The predicted functional alteration between AMO and control group. **(A)** Functional alterations of different treatment based on 16s rRNA PICRUSt analysis KEGG (level1, *P*<0.01) ; **(B)** Network of microbiota and KEGG (level3) human disease-related pathway. The different color of nodes represented different types of human disease. The size of node was set by the number of connections. The edge represented the correlation (R>0.9 or R<-0.9, *P*<0.05).

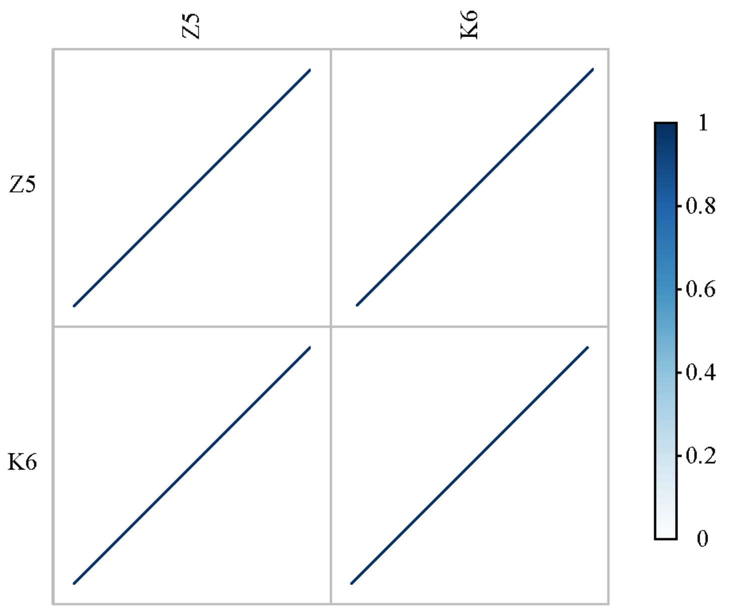

FIGURE S2 Heatmap of average nucleotide identity (ANI) distance between *K. variicola* K6 and Z5.

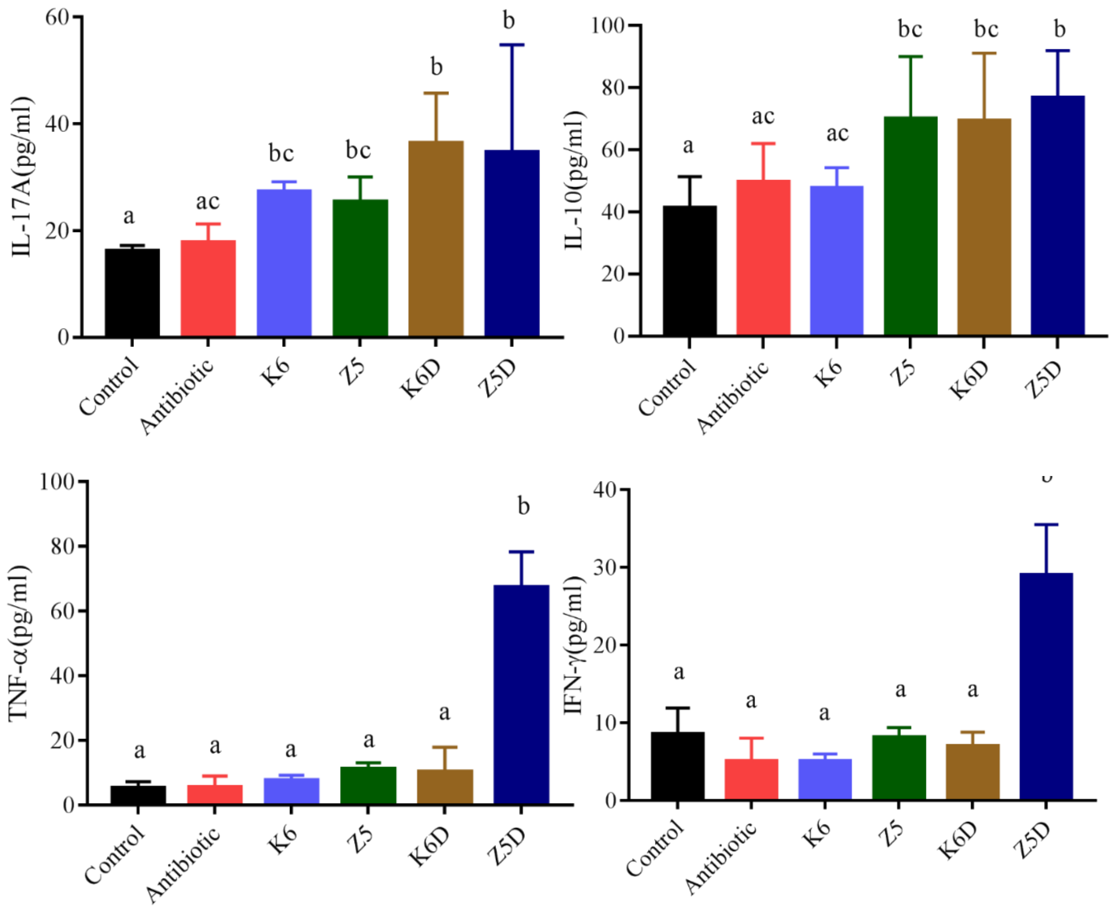

FIGURE S3 Effect of AMO associated *K. variicola* on serum cytokine levels in ABx-treated or DSS-induced colitis model mice.

Quantification of serum concentration of IL-17a, IL-10, IFN-γ and TNF-α. Different letters indicate significant differences (P<0.05, one-way ANOVA) between different groups (mean ± SD, n=3).

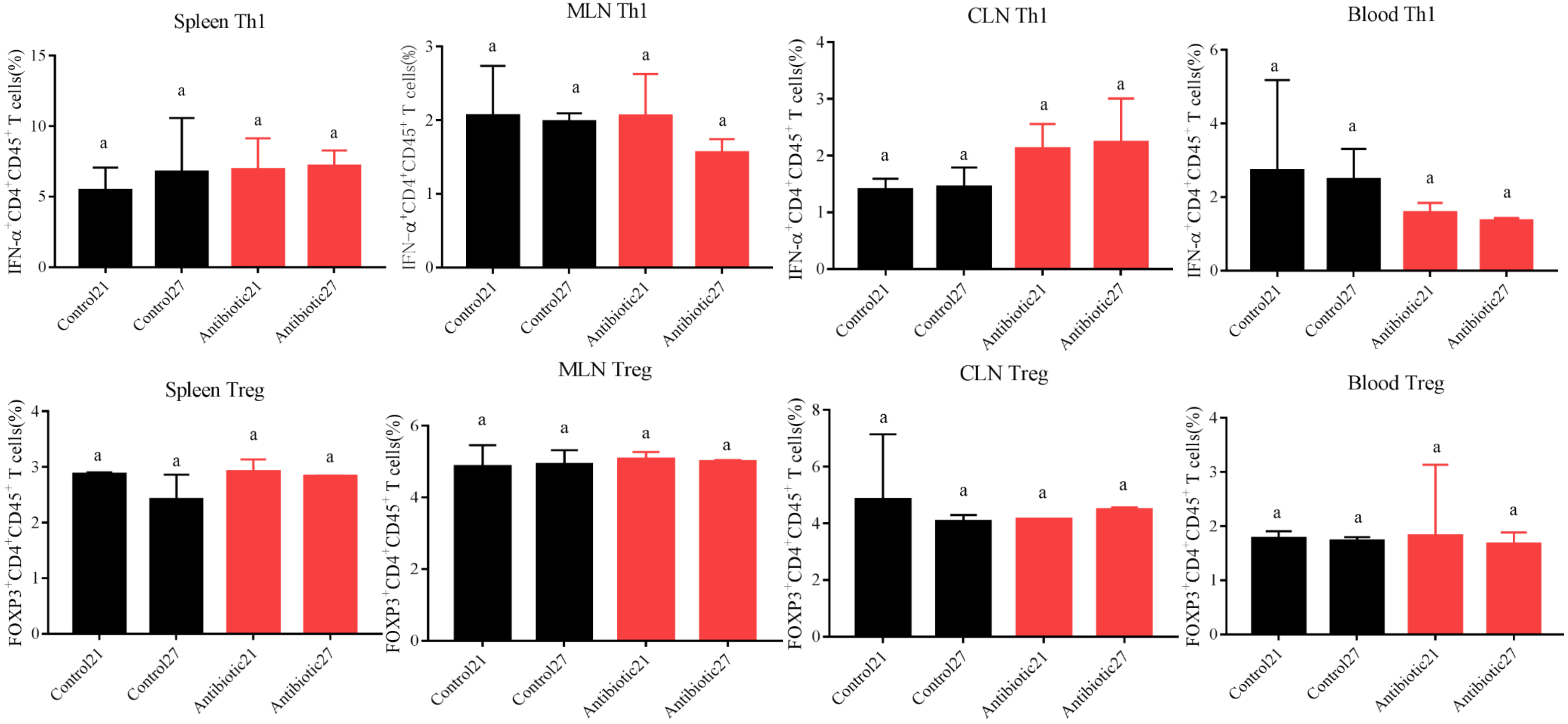

FIGURE.S4 Th1 and Treg cell variance between control and antibiotic group along with K6, Z5,K6D and Z5D euthanased. Quantification of Th1 and Treg cell proportion of spleen, MLN, CLN and blood in control and antibiotic at end of the K6, Z5 (at 21 days) and K6D, Z5D (at 27 days) treat. Different letters indicate significant differences ($P<0.05$, one-way ANOVA) between different groups (mean ± SD, n≥2).

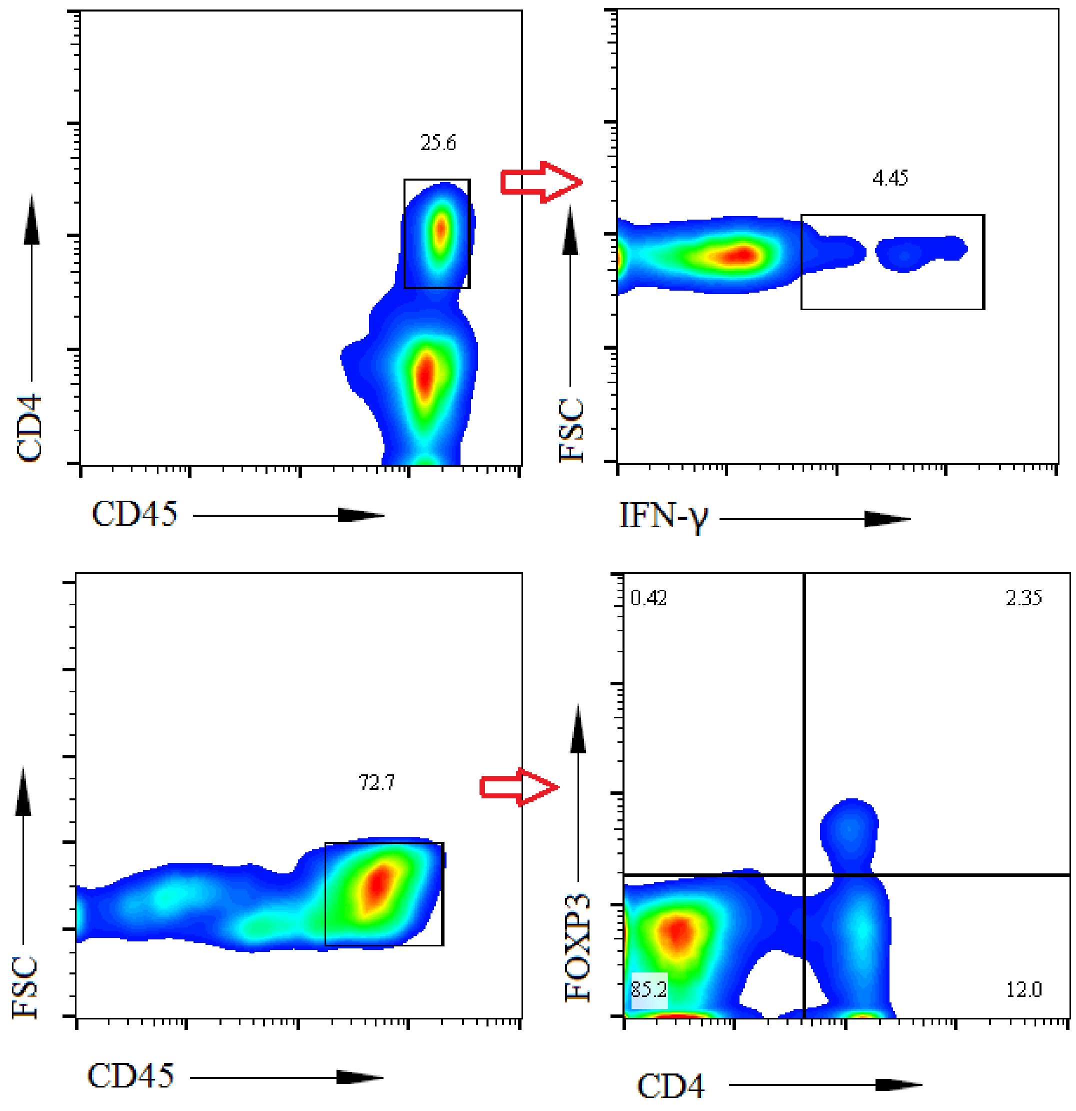

FIGURE S5 Gated CD45$^+$CD4$^+$IFN-γ$^+$ Th1 and CD45$^+$CD4$^+$FOXP3$^+$ Tregs in spleens, MLNs, CLNs and blood in each group.

SUPPLEMENT TABLES

TABLE S1 Representative sequence of of *K. variicola*.

Full-length sequences of the 16S rRNA gene from *K. variicola* strains Z5, Z7, K6 and K10 isolated from both AMO and control groups. The OUT210 and OTU680 sequence are representative sequence of these two group annotated to *Klebsiella*.

Name	Sequence	Source
K.variicola K6	AAGTTTGGTAGCGCCCTCCCGAAGGTTAAGCTACCTACTTCTTTTGCAACCCACTCCCATGGTGTGACGGGCG<br>GTGTGTACAAGGCCCGGGAACGTATTCACCGTAGCATTCTGATCTACGATTACTAGCGATTCCGACTTCATGG<br>AGTCGAGTTGCAGACTCCAATCCGGACTACGACATACTTTATGAGGTCCGCTTGCTCTCGCGAGGTCGCTTCT<br>CTTTGTATATGCCATTGTAGCACGTGTGTAGCCCTGGTCGTAAGGGCCATGATGACTTGACGTCATCCCCACCT<br>TCCTCCAGTTTATCACTGGCAGTCTCCTTTGAGTTCCCGGCCTAACCGCTGGCAACAAAGGATAAGGGTTGCG<br>CTCGTTGCGGGACTTAACCCAACATTTCACAACACGAGCTGACGACAGCCATGCAGCACCTGTCTCACAGTTC<br>CCGAAGGCACCAAAGCATCTCTGCTAAGTTCTGTGGATGTCAAGACCAGGTAAGGTTCTTCGCGTTGCATCGA<br>ATTAAACCACATGCTCCACCGCTTGTGCGGGCCCCCGTCAATTCATTTGAGTTTTAACCTTGCGGCCGTACTCC<br>CCAGGCGGTCGATTTAACGCGTTAGCTCCGGAAGCCACGCCTCAAGGGCACAACCTCCAAATCGACATCGTTT<br>ACAGCGTGGACTACCAGGGTATCTAATCCTGTTTGCTCCCCACGCTTTCGCACCTGAGCGTCAGTCTTTGTCCA<br>GGGGGCCGCCTTCGCCACCGGTATTCCTCCAGATCTCTACGCATTTCACCGCTACACCTGGAATTCTACCCCCC<br>TCTACAAGACTCTAGCCTGCCAGTTTCGAATGCAGTTCCCAGGTTGAGCCCGGGGATTTCACATCCGACTTGA<br>CAGACCGCCTGCGTGCGCTTTACGCCCAGTAATTCCGATTAACGCTTGCACCCTCCGTATTACCGCGGCTGCT<br>GGCACGGAGTTAGCCGGTGCTTCTTCTGCGGGTAACGTCAATCGACAAGGTTATTAACCTCACCGCCTTCCTC<br>CCCGCTGAAAGTGCTTTACAACCCGAAGGCCTTCTTCACACACGCGGCATGGCTGCATCAGGCTTGCGCCCAT<br>TGTGCAATATTCCCCACTGCTGCCTCCCGTAGGAGTCTGGACCGTGTCTCAGTTCCAGTGTGGCTGGTCATCCT<br>CTCAGACCAGCTAGGGATCGTCGCCTAGGTGAGCCGTTACCCCACCTACCAGCTAATCCCATCTGGGCACATC<br>TGATGGCATGAGGCCCGAAGGTCCCCCACTTTGGTCTTGCGACGTTATGCGGTATTAGCTACCGTTTCCAGTA<br>GTTATCCCCCTCCATCAGGCAGTTTCCCAGACATTACTCACCCGTCCGCCGCTCGTCACCCGAGAGCAAGCTC<br>TCTGTGCTACCGCTCGACTTGCATGTGTTAGTGCCCC	Control
K.variicola K10	TCAAAAAGTGGTAGCGCCTCCCGAAGGTTAAGCTACCTACTTCTTTTGCAACCCACTCCCATGGTGTGACGGG<br>CGGTGTGTACAAGGCCCGGGAACGTATTCACCGTAGCATTCTGATCTACGATTACTAGCGATTCCGACTTCAT<br>GGAGTCGAGTTGCAGACTCCAATCCGGACTACGACATACTTTATGAGGTCCGCTTGCTCTCGCGAGGTCGCTT<br>CTCTTTGTATATGCCATTGTAGCACGTGTGTAGCCCTGGTCGTAAGGGCCATGATGACTTGACGTCATCCCCAC<br>CTTCCTCCAGTTTATCACTGGCAGTCTCCTTTGAGTTCCCGGCCTAACCGCTGGCAACAAAGGATAAGGGTTG<br>CGCTCGTTGCGGGACTTAACCCAACATTTCACAACACGAGCTGACGACAGCCATGCAGCACCTGTCTCACAGT<br>TCCCGAAGGCACCAAAGCATCTCTGCTAAGTTCTGTGGATGTCAAGACCAGGTAAGGTTCTTCGCGTTGCATC<br>GAATTAAACCACATGCTCCACCGCTTGTGCGGGCCCCCGTCAATTCATTTGAGTTTTAACCTTGCGGCCGTACT<br>CCCCAGGCGGTCGATTTAACGCGTTAGCTCCGGAAGCCACGCCTCAAGGGCACAACCTCCAAATCGACATCG<br>TTTACAGCGTGGACTACCAGGGTATCTAATCCTGTTTGCTCCCCACGCTTTCGCACCTGAGCGTCAGTCTTTGT	Control

	CCAGGGGGCCGCCTTCGCCACCGGTATTCCTCCAGATCTCTACGCATTTCACCGCTACACCTGGAATTCTACCC<br>CCCTCTACAAGACTCTAGCCTGCCAGTTTCGAATGCAGTTCCCAGGTTGAGCCCGGGGATTTCACATCCGACT<br>TGACAGACCGCCTGCGTGCGCTTTACGCCCAGTAATTCCGATTAACGCTTGCACCCTCCGTATTACCGCGGCT<br>GCTGGCACGGAGTTAGCCGGTGCTTCTTCTGCGGGTAACGTCAATCGACAAGGTTATTAACCTCACCGCCTTC<br>CTCCCCGCTGAAAGTGCTTTACAACCCGAAGGCCTTCTTCACACACGCGGCATGGCTGCATCAGGCTTGCGCC<br>CATTGTGCAATATTCCCCACTGCTGCCTCCCGTAGGAGTCTGGACCGTGTCTCAGTTCCAGTGTGGCTGGTCAT<br>CCTCTCAGACCAGCTAGGGATCGTCGCCTAGGTGAGCCGTTACCCCACCTACCAGCTAATCCCATCTGGGCAC<br>ATCTGATGGCATGAGGCCCGAAGGTCCCCCACTTTGGTCTTGCGACGTTATGCGGTATTAGCTACCGTTTCCA<br>GTAGTTATCCCCCTCCATCAGGCAGTTTCCCAGACATTACTCACCCGTCCGCCGCTCGTCACCCGAGAGCAAG<br>CTCTCTGTGCTACCGCTCGACTGCATGTGTAGGCCTGCACCCT	
K.variicola Z5	TGGGGTTAAGCGCCCTCCCGAAGGTTAAGCTACCTACTTCTTTTGCAACCCACTCCCATGGTGTGACGGGCGG<br>TGTGTACAAGGCCCGGGAACGTATTCACCGTAGCATTCTGATCTACGATTACTAGCGATTCCGACTTCATGGA<br>GTCGAGTTGCAGACTCCAATCCGGACTACGACATACTTTATGAGGTCCGCTTGCTCTCGCGAGGTCGCTTCTCT<br>TTGTATATGCCATTGTAGCACGTGTGTAGCCCTGGTCGTAAGGGCCATGATGACTTGACGTCATCCCCACCTTC<br>CTCCAGTTTATCACTGGCAGTCTCCTTTGAGTTCCCGGCCTAACCGCTGGCAACAAAGGATAAGGGTTGCGCT<br>CGTTGCGGGACTTAACCCAACATTTCACAACACGAGCTGACGACAGCCATGCAGCACCTGTCTCACAGTTCCC<br>GAAGGCACCAAAGCATCTCTGCTAAGTTCTGTGGATGTCAAGACCAGGTAAGGTTCTTCGCGTTGCATCGAAT<br>TAAACCACATGCTCCACCGCTTGTGCGGGCCCCCGTCAATTCATTTGAGTTTTAACCTTGCGGCCGTACTCCCC<br>AGGCGGTCGATTTAACGCGTTAGCTCCGGAAGCCACGCCTCAAGGGCACAACCTCCAAATCGACATCGTTTAC<br>AGCGTGGACTACCAGGGTATCTAATCCTGTTTGCTCCCCACGCTTTCGCACCTGAGCGTCAGTCTTTGTCCAGG<br>GGGCCGCCTTCGCCACCGGTATTCCTCCAGATCTCTACGCATTTCACCGCTACACCTGGAATTCTACCCCCCTC<br>TACAAGACTCTAGCCTGCCAGTTTCGAATGCAGTTCCCAGGTTGAGCCCGGGGATTTCACATCCGACTTGACA<br>GACCGCCTGCGTGCGCTTTACGCCCAGTAATTCCGATTAACGCTTGCACCCTCCGTATTACCGCGGCTGCTGG<br>CACGGAGTTAGCCGGTGCTTCTTCTGCGGGTAACGTCAATCGACAAGGTTATTAACCTCACCGCCTTCCTCCC<br>CGCTGAAAGTGCTTTACAACCCGAAGGCCTTCTTCACACACGCGGCATGGCTGCATCAGGCTTGCGCCCATTG<br>TGCAATATTCCCCACTGCTGCCTCCCGTAGGAGTCTGGACCGTGTCTCAGTTCCAGTGTGGCTGGTCATCCTCT<br>CAGACCAGCTAGGGATCGTCGCCTAGGTGAGCCGTTACCCCACCTACCAGCTAATCCCATCTGGGCACATCTG<br>ATGGCATGAGGCCCGAAGGTCCCCCACTTTGGTCTTGCGACGTTATGCGGTATTAGCTACCGTTTCCAGTAGT<br>TATCCCCCTCCATCAGGCAGTTTCCCAGACATTACTCACCCGTCCGCCGCTCGTCACCCGAGAGCAAGCTCTCT<br>GTGCTACCGCTCGACTTGCATGTGTAAGT	AMO

K.variicola Z7	TTAAGTTGGTAGCGCCCTCCCGAAGGTTAAGCTACCTACTTCTTTTGCAACCCACTCCCATGGTGTGACGGGC<br>GGTGTGTACAAGGCCCGGGAACGTATTCACCGTAGCATTCTGATCTACGATTACTAGCGATTCCGACTTCATG<br>GAGTCGAGTTGCAGACTCCAATCCGGACTACGACATACTTTATGAGGTCCGCTTGCTCTCGCGAGGTCGCTTC<br>TCTTTGTATATGCCATTGTAGCACGTGTGTAGCCCTGGTCGTAAGGGCCATGATGACTTGACGTCATCCCCACC<br>TTCCTCCAGTTTATCACTGGCAGTCTCCTTTGAGTTCCCGGCCTAACCGCTGGCAACAAAGGATAAGGGTTGC<br>GCTCGTTGCGGGACTTAACCCAACATTTCACAACACGAGCTGACGACAGCCATGCAGCACCTGTCTCACAGTT<br>CCCGAAGGCACCAAAGCATCTCTGCTAAGTTCTGTGGATGTCAAGACCAGGTAAGGTTCTTCGCGTTGCATCG<br>AATTAAACCACATGCTCCACCGCTTGTGCGGGCCCCCGTCAATTCATTTGAGTTTTAACCTTGCGGCCGTACTC<br>CCCAGGCGGTCGATTTAACGCGTTAGCTCCGGAAGCCACGCCTCAAGGGCACAACCTCCAAATCGACATCGTT<br>TACAGCGTGGACTACCAGGGTATCTAATCCTGTTTGCTCCCCACGCTTTCGCACCTGAGCGTCAGTCTTTGTCC<br>AGGGGGCCGCCTTCGCCACCGGTATTCCTCCAGATCTCTACGCATTTCACCGCTACACCTGGAATTCTACCCCC<br>CTCTACAAGACTCTAGCCTGCCAGTTTCGAATGCAGTTCCCAGGTTGAGCCCGGGGATTTCACATCCGACTTG<br>ACAGACCGCCTGCGTGCGCTTTACGCCCAGTAATTCCGATTAACGCTTGCACCCTCCGTATTACCGCGGCTGC<br>TGGCACGGAGTTAGCCGGTGCTTCTTCTGCGGGTAACGTCAATCGACAAGGTTATTAACCTCACCGCCTTCCT<br>CCCCGCTGAAAGTGCTTTACAACCCGAAGGCCTTCTTCACACACGCGGCATGGCTGCATCAGGCTTGCGCCCA<br>TTGTGCAATATTCCCCACTGCTGCCTCCCGTAGGAGTCTGGACCGTGTCTCAGTTCCAGTGTGGCTGGTCATCC<br>TCTCAGACCAGCTAGGGATCGTCGCCTAGGTGAGCCGTTACCCCACCTACCAGCTAATCCCATCTGGGCACAT<br>CTGATGGCATGAGGCCCGAAGGTCCCCCACTTTGGTCTTGCGACGTTATGCGGTATTAGCTACCGTTTCCAGT<br>AGTTATCCCCCTCCATCAGGCAGTTTCCCAGACATTACTCACCCGTCCGCCGCTCGTCACCCGAGAGCAAGCT<br>CTCTGTGCTACCGCTCGACTGCATGTGTAGCTCCACAA	AMO
OTU210	CCTACGGGTGGCTGCAGTGGGGAATATTGCACAATGGGCGCAAGCCTGATGCAGCCATGCCGCGTGTGTGAA<br>GAAGGCCTTCGGGTTGTAAAGCACTTTCAGCGGGGAGGAAGGCGATAAGGTTAATAACCTTGTCGATTGACG<br>TTACCCGCAGAAGAAGCACCGGCTAACTCCGTGCCAGCAGCCGCGGTAATACGGAGGGTGCAAGCGTTAATC<br>GGAATTACTGGGCGTAAAGCGCACGCAGGCGGTCTGTCAAGTCGGATGTGAAATCCCCGGGCTCAACCTGGG<br>AACTGCATTCGAAACTGGCAGGCTAGAGTCTTGTAGAGGGGGGTAGAATTCCAGGTGTAGCGGTGAAATGCG<br>TAGAGATCTGGAGGAATACCGGTGGCGAAGGCGGCCCCCTGGACAAAGACTGACGCTCAGGTGCGAAAGCG<br>TGGGGAGCAAACAGGATTAGATACCCTAGTAGTCC	AMO,<br>Control
OTU680	GTTGAAACTCCTACGGGAGGCAGCAGTGGGGAATATTGCACAATGGGGGAAACCCTGATGCAGCGACGCCGC<br>GTGAGTGATGAAGTATTTCGGTATGTAAAGCTCTATCAGCAGGGAAGATAATGACGGTACCTGACTAAGAAG<br>CTCCGGCTAAATACGTGCCAGCAGCCGCGGTAATACGTATGGAGCAAGCGTTATCCGGATTTACTGGGTGTAA<br>AGGGAGCGCAGACGGCAGGGCAAGTCTGATGTGAAAGTCCGGGGCTCAACCCCGGGACTGCATTGGAAACT	AMO,<br>Control

GTCCGGCTAGAGTGCAGGAGAGGTAAGTGGAATTCCTAGTGTAGCGGTGAAATGCGTAGATATTAGGAGGAA
CACCAGTGGCGAAGGCGGCTTACTGGACTGTAACTGACGTTGAGGCTCGAAAGCGTGGGGAGCAAACAGGAT
TAGATACCCCGGTAGTCCACGATG

TABLE S2 Strains identification of colonies isolated from both control and AMO group.

Identification results of randomly picked 28 colonies isolated from both AMO and control groups (3 samples respectively).

Sample	Group	Number	Colonies Name	Description	Accession
SampleA1	AMO	1	K1	*Klebsiella pneumonia*	GQ166863.1
		2	K2	*Acinetobacter baumannii*	MG554737.1
		3	K3	*Esherichia coli*	DQ819196.1
		4	K4	*Klebsiella variicola*	JN848785.1
		5	K5	*Klebsiella variicola*	JN848785.1
SmapleA2	AMO	1	Z1	*Acinetobacter baumannii*	MG554737.1
		2	Z2	*Klebsiella variicola*	JN848785.1
		3	Z3	*Esherichia coli*	MG388227.1
		4	Z4	*Esherichia coli*	EF560780.1
		5	Z5	*Klebsiella variicola*	JN848785.1
SampleA3	AMO	1	Z6	*Klebsiella variicola*	JN848785.1
		2	Z7	*Klebsiella pneumonia*	GQ166863.1
		3	Z8	*Klebsiella variicola*	JN848785.1
		4	Z9	*Klebsiella variicola*	JN848785.1
SampleC1	Control	1	K6	*Klebsiella variicola*	JN848785.1
		2	K7	*Klebsiella pneumonia*	MG388227.1
		3	K8	*Esherichia coli*	MG388227.1
		4	K9	*Klebsiella variicola*	JN848785.1
		5	K10	*Klebsiella pneumonia*	GQ166863.1

SampleC2	Control	1	Z10	*Klebsiella variicola*	JN848785.1
		2	Z11	*Esherichia coli*	HM021544.1
		3	Z12	*Serratia liquefaciens*	GU586145.1
		4	Z13	*Klebsiella variicola*	JN848785.1
		5	Z14	*Klebsiella pneumonia*	GQ166863.1
SampleC3	Control	1	Z15	*Klebsiella variicola*	JN848785.1
		2	Z16	*Klebsiella variicola*	JN848785.1
		3	Z17	*Klebsiella variicola*	JN848785.1
		4	Z18	*Acinetobacter baumannii*	MG554737.1

TABLE S3 Representative sequences of the colonies isolated from control and AMO groups.

Description	Accession	Representative sequence
Klebsiella penumoniae	GQ166863.1	GGTCAATCAAAGTGGTAGCGCCCTCCGAAGGTTAAGCTACCTACTTCTTTTGCAACCCACTCCCATGGTGTG<br>ACGGGCGGTGTGTACAAGGCCCGGGAACGTATTCACCGTAGCATTCTGATCTACGATTACTAGCGATTCCG<br>ACTTCATGGAGTCGAGTTGCAGACTCCAATCCGGACTACGACATACTTTATGAGGTCCGCTTGCTCTCGCGA<br>GGTCGCTTCTCTTTGTATATGCCATTGTAGCACGTGTGTAGCCCTGGTCGTAAGGGCCATGATGACTTGACG<br>TCATCCCCACCTTCCTCCAGTTTATCACTGGCAGTCTCCTTTGAGTTCCCGGCCTAACCGCTGGCAACAAAG<br>GATAAGGGTTGCGCTCGTTGCGGGACTTAACCCAACATTTCACAACACGAGCTGACGACAGCCATGCAGCA<br>CCTGTCTCACAGTTCCCGAAGGCACCAAAGCATCTCTGCTAAGTTCTGTGGATGTCAAGACCAGGTAAGGT<br>TCTTCGCGTTGCATCGAATTAAACCACATGCTCCACCGCTTGTGCGGGCCCCCGTCAATTCATTTGAGTTTT<br>AACCTTGCGGCCGTACTCCCCAGGCGGTCGATTTAACGCGTTAGCTCCGGAAGCCACGCCTCAAGGGCACA<br>ACCTCCAAATCGACATCGTTTACAGCGTGGACTACCAGGGTATCTAATCCTGTTTGCTCCCCACGCTTTCGC<br>ACCTGAGCGTCAGTCTTTGTCCAGGGGGCCGCCTTCGCCACCGGTATTCCTCCAGATCTCTACGCATTTCAC<br>CGCTACACCTGGAATTCTACCCCCCTCTACAAGACTCTAGCCTGCCAGTTTCGAATGCAGTTCCCAGGTTGA<br>GCCCGGGGATTTCACATCCGACTTGACAGACCGCCTGCGTGCGCTTACGCCCAGTAATTCCGATTAACGCTT<br>GCACCCTCCGTATTACCGCGGCTGCTGGCACGGAGTTAGCCGGTGCTTCTTCTGCGGGTAACGTCAATCGAC<br>AAGGTTATTAACCTCACCGCCTTCCTCCCCGCTGAAAGTGCTTTACAACCCGAAGGCCTTCTTCACACACGC<br>GGCATGGCTGCATCAGGCTTGCGCCCATTGTGCAATATTCCCCACTGCTGCCTCCCGTAGGAGTCTGGACCG<br>TGTCTCAGTTCCAGTGTGGCTGGTCATCCTCTCAGACCAGCTAGGGATCGTCGCCTAGGTGAGCCGTTACCC<br>CACCTACCAGCTAATCCCATCTGGGCACATCTGATGGCATGAGGCCCGAAGGTCCCCCACTTTGGTCTTGCG<br>ACGTTATGCGGTATTAGCTACCGTTTCCAGTAGTTATCCCCCTCCATCAGGCAGTTTCCCAGACATTACTCA<br>CCCGTCCGCCGCTCGTCACCCGAGAGCAAGCTCTCTGTGCTACCGCTCGACTGCATGTGTAGGCTCCCCCGC
Acinetobacter baumannii	MG554737.1	GAAGGTTGGGTAAGCGCCCTCCGAGGTTAAGCTACCTACTTCTTTTGCAACCCACTCCCATGGTGTGACGGG<br>CGGTGTGTACAAGGCCCGGGAACGTATTCACCGTGGCATTCTGATCCACGATTACTAGCGATTCCGACTTCA<br>TGGAGTCGAGTTGCAGACTCCAATCCGGACTACGACGCACTTTATGAGGTCCGCTTGCTCTCGCGAGGTCG<br>CTTCTCTTTGTATGCGCCATTGTAGCACGTGTGTAGCCCTGGTCGTAAGGGCCATGATGACTTGACGTCATC<br>CCCACCTTCCTCCAGTTTATCACTGGCAGTCTCCTTTGAGTTCCCGGCCGGACCGCTGGCAACAAAGGATAA<br>GGGTTGCGCTCGTTGCGGGACTTAACCCAACATTTCACAACACGAGCTGACGACAGCCATGCAGCACCTGT<br>CTCACGGTTCCCGAAGGCACATTCTCATCTCTGAAAACTTCCGTGGATGTCAAGACCAGGTAAGGTTCTTCG<br>CGTTGCATCGAATTAAACCACATGCTCCACCGCTTGTGCGGGCCCCCGTCAATTCATTTGAGTTTTAACCTT<br>GCGGCCGTACTCCCCAGGCGGTCGACTTAACGCGTTAGCTCCGGAAGCCACGCCTCAAGGGCACAACCTCC<br>AAGTCGACATCGTTTACGGCGTGGACTACCAGGGTATCTAATCCTGTTTGCTCCCCACGCTTTCGCACCTGA

		GCGTCAGTCTTCGTCCAGGGGGCCGCCTTCGCCACCGGTATTCCTCCAGATCTCTACGCATTTCACCGCTAC<br>ACCTGGAATTCTACCCCCCTCTACGAGACTCAAGCTTGCCAGTATCAGATGCAGTTCCCAGGTTGAGCCCG<br>GGGATTTCACATCTGACTTAACAAACCGCCTGCGTGCGCTTTACGCCCAGTAATTCCGATTAACGCTTGCAC<br>CCTCCGTATTACCGCGGCTGCTGGCACGGAGTTAGCCGGTGCTTCTTCTGCGGGTAACGTCAATGAGCAAA<br>GGTATTAACTTTACTCCCTTCCTCCCCGCTGAAAGTACTTTACAACCCGAAGGCCTTCTTCATACACGCGGC<br>ATGGCTGCATCAGGCTTGCGCCCATTGTGCAATATTCCCCACTGCTGCCTCCCGTAGGAGTCTGGACCGTGT<br>CTCAGTTCCAGTGTGGCTGGTCATCCTCTCAGACCAGCTAGGGATCGTCGCCTAGGTGAGCCGTTACCCCAC<br>CTACTAGCTAATCCCATCTGGGCACATCCGATGGCAAGAGGCCCGAAGGTCCCCCTCTTTGGTCTTGCGACG<br>TTATGCGGTATTAGCTACCGTTTCCAGTAGTTATCCCCCTCCATCAGGCAGTTTCCCAGACATTACTCACCC<br>GTCCGCCACTCGTCAGCGAAACAGCAAGCTGTTTCCTGTTACCGTTCGACTGCATGTGTAGACTGCCCGAG
Escherichia coli	DQ819196.1	AAAAGTTGGGGTAAGGCGCCCTCCCGAGGTTAAGCTACCTACTTCTTTTCAACCCACTCCCATGGTGTGACG<br>GGCGGTGTGTACAAGGCCCGGGAACGTATTCACCGTGGCATTCTGATCCACGATTACTAGCGATTCCGACT<br>TCATGGAGTCGAGTTGCAGACTCCAATCCGGACTACGACGCACTTTATGAGGTCCGCTTGCTCTCGCGAGGT<br>CGCTTCTCTTTGTATGCGCCATTGTAGCACGTGTGTAGCCCTGGTCGTAAGGGCCATGATGACTTGACGTCA<br>TCCCCACCTTCCTCCAGTTTATCACTGGCAGTCTCCTTTGAGTTCCCGGCCGGACCGCTGGCAACAAAGGAT<br>AAGGGTTGCGCTCGTTGCGGGACTTAACCCAACATTTCACAACACGAGCTGACGACAGCCATGCAGCACCT<br>GTCTCACGGTTCCCGAAGGCACATTCTCATCTCTGAAAACTTCCGTGGATGTCAAGACCAGGTAAGGTTCTT<br>CGCGTTGCATCGAATTAAACCACATGCTCCACCGCTTGTGCGGGCCCCCGTCAATTCATTTGAGTTTTAACC<br>TTGCGGCCGTACTCCCCAGGCGGTCGACTTAACGCGTTAGCTCCGGAAGCCACGCCTCAAGGGCACAACCT<br>CCCAAGTCGACATCGTTTACGGCGTGGACTACCAGGGTATCTAATCCTGTTTGCTCCCCACGCTTTCGCACC<br>TGAGCGTCAGTCTTCGTCCAGGGGGCCGCCTTCGCCACCGGTATTCCTCCAGATCTCTACGCATTTCACCGC<br>TACACCTGGAATTCTACCCCCCTCTACGAGACTCAAGCTTGCCAGTATCAGATGCAGTTCCCAGGTTGAGCC<br>CGGGGATTTCACATCTGACTTAACAAACCGCCTGCGTGCGCTTTACGCCCAGTAATTCCGATTAACGCTTGC<br>ACCCTCCGTATTACCGCGGCTGCTGGCACGGAGTTAGCCGGTGCTTCTTCTGCGGGTAACGTCAATGAGCA<br>AAGGTATTAACTTTACTCCCTTCCTCCCCGCTGAAAGTACTTTACAACCCGAAGGCCTTCTTCATACACGCG<br>GCATGGCTGCATCAGGCTTGCGCCCATTGTGCAATATTCCCCACTGCTGCCTCCCGTAGGAGTCTGGACCGT<br>GTCTCAGTTCCAGTGTGGCTGGTCATCCTCTCAGACCAGCTAGGGATCGTCGCCTAGGTGAGCCGTTACCCC<br>ACCTACTAGCTAATCCCATCTGGGCACATCCGATGGCAAGAGGCCCGAAGGTCCCCCTCTTTGGTCTTGCGA<br>CGTTATGCGGTATTAGCTACCGTTTCCAGTAGTTATCCCCCTCCATCAGGCAGTTTCCCAGACATTACTCAC<br>CCGTCCGCCACTCGTCAGCGAAACAGCAAGCTGTTTCCTGTTACCGTTCGACTGCATGTGTAGTCTCCCCCC

Klebsiella variicola	JN848785.1	TAGGAGGTGGTAAGCGCCCTCCCGAAGGTTAAGCTACCTACTTCTTTTGCAACCCACTCCCATGGTGTGACG<br>GGCGGTGTGTACAAGGCCCGGGAACGTATTCACCGTAGCATTCTGATCTACGATTACTAGCGATTCCGACTT<br>CATGGAGTCGAGTTGCAGACTCCAATCCGGACTACGACATACTTTATGAGGTCCGCTTGCTCTCGCGAGGTC<br>GCTTCTCTTTGTATATGCCATTGTAGCACGTGTGTAGCCCTGGTCGTAAGGGCCATGATGACTTGACGTCAT<br>CCCCACCTTCCTCCAGTTTATCACTGGCAGTCTCCTTTGAGTTCCCGGCCTAACCGCTGGCAACAAAGGATA<br>AGGGTTGCGCTCGTTGCGGGACTTAACCCAACATTTCACAACACGAGCTGACGACAGCCATGCAGCACCTG<br>TCTCACAGTTCCCGAAGGCACCAAAGCATCTCTGCTAAGTTCTGTGGATGTCAAGACCAGGTAAGGTTCTTC<br>GCGTTGCATCGAATTAAACCACATGCTCCACCGCTTGTGCGGGCCCCCGTCAATTCATTTGAGTTTTAACCT<br>TGCGGCCGTACTCCCCAGGCGGTCGATTTAACGCGTTAGCTCCGGAAGCCACGCCTCAAGGGCACAACCTC<br>CAAATCGACATCGTTTACAGCGTGGACTACCAGGGTATCTAATCCTGTTTGCTCCCCACGCTTTCGCACCTG<br>AGCGTCAGTCTTTGTCCAGGGGGCCGCCTTCGCCACCGGTATTCCTCCAGATCTCTACGCATTTCACCGCTA<br>CACCTGGAATTCTACCCCCCTCTACAAGACTCTAGCCTGCCAGTTTCGAATGCAGTTCCCAGGTTGAGCCCG<br>GGGATTTCACATCCGACTTGACAGACCGCCTGCGTGCGCTTTACGCCCAGTAATTCCGATTAACGCTTGCAC<br>CCTCCGTATTACCGCGGCTGCTGGCACGGAGTTAGCCGGTGCTTCTTCTGCGGGTAACGTCAATCGACAAG<br>GTTATTAACCTCACCGCCTTCCTCCCCGCTGAAAGTGCTTTACAACCCGAAGGCCTTCTTCACACACGCGGC<br>ATGGCTGCATCAGGCTTGCGCCCATTGTGCAATATTCCCCACTGCTGCCTCCCGTAGGAGTCTGGACCGTGT<br>CTCGTTCCAGTGTGGCTGGTCATCCTCTCAGACCAGCTAGGGATCGTCGCCTAGGTGAGCCGTTACCCCACC<br>TACCAGCTAATCCCATCTGGGCACATCTGATGGCATGAGGCCCGAAGGTCCCCCACTTTGGTCTTGCGACGT<br>TATGCGGTATTAGCTACCGTTTCCAGTAGTTATCCCCCTCCATCAGGCAGTTTCCCAGACATTACTCACCCG<br>TCCGCCGCTCGTCACCCGAGAGCAAGCTCTCTGTGCTACCGCTCGACTGCATGTGTAGACACC
Escherichia coli	MG388227.1	AAAAGTTGGGGTAAGGCGCCCTCCCGAGGTTAAGCTACCTACTTCTTTTGCAACCCACTCCCATGGTGTGAC<br>GGGCGGTGTGTACAAGGCCCGGGAACGTATTCACCGTGGCATTCTGATCCACGATTACTAGCGATTCCGAC<br>TTCATGGAGTCGAGTTGCAGACTCCAATCCGGACTACGACGCACTTTATGAGGTCCGCTTGCTCTCGCGAGG<br>TCGCTTCTCTTTGTATGCGCCATTGTAGCACGTGTGTAGCCCTGGTCGTAAGGGCCATGATGACTTGACGTC<br>ATCCCCACCTTCCTCCAGTTTATCACTGGCAGTCTCCTTTGAGTTCCCGGCCGGACCGCTGGCAACAAAGGA<br>TAAGGGTTGCGCTCGTTGCGGGACTTAACCCAACATTTCACAACACGAGCTGACGACAGCCATGCAGCACC<br>TGTCTCACGGTTCCCGAAGGCACATTCTCATCTCTGAAAACTTCCGTGGATGTCAAGACCAGGTAAGGTTCT<br>TCGCGTTGCATCGAATTAAACCACATGCTCCACCGCTTGTGCGGGCCCCCGTCAATTCATTTGAGTTTTAAC<br>CTTGCGGCCGTACTCCCCAGGCGGTCGACTTAACGCGTTAGCTCCGGAAGCCACGCCTCAAGGGCACAACC<br>TCCCAAGTCGACATCGTTTACGGCGTGGACTACCAGGGTATCTAATCCTGTTTGCTCCCCACGCTTTCGCAC<br>CTGAGCGTCAGTCTTCGTCCAGGGGGCCGCCTTCGCCACCGGTATTCCTCCAGATCTCTACGCATTTCACCG

		CTACACCTGGAATTCTACCCCCCTCTACGAGACTCAAGCTTGCCAGTATCAGATGCAGTTCCCAGGTTGAGC<br>CCGGGGATTTCACATCTGACTTAACAAACCGCCTGCGTGCGCTTTACGCCCAGTAATTCCGATTAACGCTTG<br>CACCCTCCGTATTACCGCGGCTGCTGGCACGGAGTTAGCCGGTGCTTCTTCTGCGGGTAACGTCAATGAGCA<br>AAGGTATTAACTTTACTCCCTTCCTCCCCGCTGAAAGTACTTTACAACCCGAAGGCCTTCTTCATACACGCG<br>GCATGGCTGCATCAGGCTTGCGCCCATTGTGCAATATTCCCCACTGCTGCCTCCCGTAGGAGTCTGGACCGT<br>GTCTCAGTTCCAGTGTGGCTGGTCATCCTCTCAGACCAGCTAGGGATCGTCGCCTAGGTGAGCCGTTACCCC<br>ACCTACTAGCTAATCCCATCTGGGCACATCCGATGGCAAGAGGCCCGAAGGTCCCCCTCTTTGGTCTTGCGA<br>CGTTATGCGGTATTAGCTACCGTTTCCAGTAGTTATCCCCCTCCATCAGGCAGTTTCCCAGACATTACTCAC<br>CCGTCCGCCACTCGTCAGCGAAACAGCAAGCTGTTTCCTGTTACCGTTCGACTGCATGTGTAGTCTCCCCCC
Escherichia coli	EF560780.1	TTAGTACCAAGTGAGTAGCGCCCTCCCGAGGTTAAGCTACCTACTTCTTTTGCAACCCACTCCCATGGTGTG<br>ACGGGCGGTGTGTACAAGGCCCGGGAACGTATTCACCGTGGCATTCTGATCCACGATTACTAGCGATTCCG<br>ACTTCATGGAGTCGAGTTGCAGACTCCAATCCGGACTACGACGCACTTTATGAGGTCCGCTTGCTCTCGCGA<br>GGTCGCTTCTCTTTGTATGCGCCATTGTAGCACGTGTGTAGCCCTGGTCGTAAGGGCCATGATGACTTGACG<br>TCATCCCCACCTTCCTCCAGTTTATCACTGGCAGTCTCCTTTGAGTTCCCGGCCGGACCGCTGGCAACAAAG<br>GATAAGGGTTGCGCTCGTTGCGGGACTTAACCCAACATTTCACAACACGAGCTGACGACAGCCATGCAGCA<br>CCTGTCTCACGGTTCCCGAAGGCACATTCTCATCTCTGAAAACTTCCGTGGATGTCAAGACCAGGTAAGGTT<br>CTTCGCGTTGCATCGAATTAAACCACATGCTCCACCGCTTGTGCGGGCCCCCGTCAATTCATTTGAGTTTTA<br>ACCTTGCGGCCGTACTCCCCAGGCGGTCGACTTAACGCGTTAGCTCCGGAAGCCACGCCTCAAGGGCACAA<br>CCTCCAAGTCGACATCGTTTACGGCGTGGACTACCAGGGTATCTAATCCTGTTTGCTCCCCACGCTTTCGCA<br>CCTGAGCGTCAGTCTTCGTCCAGGGGGCCGCCTTCGCCACCGGTATTCCTCCAGATCTCTACGCATTTCACC<br>GCTACACCTGGAATTCTACCCCCCTCTACGAGACTCAAGCTTGCCAGTATCAGATGCAGTTCCCAGGTTGAG<br>CCCGGGGATTTCACATCTGACTTAACAAACCGCCTGCGTGCGCTTTACGCCCAGTAATTCCGATTAACGCTT<br>GCACCCTCCGTATTACCGCGGCTGCTGGCACGGAGTTAGCCGGTGCTTCTTCTGCGGGTAACGTCAATGAGC<br>AAAGGTATTAACTTTACTCCCTTCCTCCCCGCTGAAAGTACTTTACAACCCGAAGGCCTTCTTCATACACGC<br>GGCATGGCTGCATCAGGCTTGCGCCCATTGTGCAATATTCCCCACTGCTGCCTCCCGTAGGAGTCTGGACCG<br>TGTCTCAGTTCCAGTGTGGCTGGTCATCCTCTCAGACCAGCTAGGGATCGTCGCCTAGGTGAGCCGTTACCC<br>CACCTACTAGCTAATCCCATCTGGGCACATCCGATGGCAAGAGGCCCGAAGGTCCCCCTCTTTGGTCTTGCG<br>ACGTTATGCGGTATTAGCTACCGTTTCCAGTAGTTATCCCCCTCCATCAGGCAGTTTCCCAGACATTACTCA<br>CCCGTCCGCCACTCGTCAGCGAAACAGCAAGCTGT<br>TTCCTGTTACCGTTCGACTGCATGTGTAGCAGCGCCCCC

Escherichia coli	HM021544.1	TTAATCAAGGTGGTAGCGCCCTCCCGAAGGTTAAGCTACCTACTTCTTTTGCAACCCACTCCCATGGTGTGA<br>CGGGCGGTGTGTACAAGGCCCGGAACGTATTCACCGTGGCATTCTGATCCACGATTACTAGCGATTCCGA<br>CTTCATGGAGTCGAGTTGCAGACTCCAATCCGGACTACGACGCACTTTATGAGGTCCGCTTGCTCTCGCGAG<br>GTCGCTTCTCTTTGTATGCGCCATTGTAGCACGTGTGTAGCCCTGGTCGTAAGGGCCATGATGACTTGACGT<br>CATCCCCACCTTCCTCCAGTTTATCACTGGCAGTCTCCTTTGAGTTCCCGGCCGGACCGCTGGCAACAAAGG<br>ATAAGGGTTGCGCTCGTTGCGGGACTTAACCCAACATTTCACAACACGAGCTGACGACAGCCATGCAGCAC<br>CTGTCTCACGGTTCCCGAAGGCACATTCTCATCTCTGAAAACTTCCGTGGATGTCAAGACCAGGTAAGGTTC<br>TTCGCGTTGCATCGAATTAAACCACATGCTCCACCGCTTGTGCGGGCCCCCGTCAATTCATTTGAGTTTTAA<br>CCTTGCGGCCGTACTCCCCAGGCGGTCGACTTAACGCGTTAGCTCCGGAAGCCACGCCTCAAGGGCACAAC<br>CTCCAAGTCGACATCGTTTACGGCGTGGACTACCAGGGTATCTAATCCTGTTTGCTCCCCACGCTTTCGCAC<br>CTGAGCGTCAGTCTTCGTCCAGGGGGCCGCCTTCGCCACCGGTATTCCTCCAGATCTCTACGCATTTCACCG<br>CTACACCTGGAATTCTACCCCCCTCTACGAGACTCAAGCTTGCCAGTATCAGATGCAGTTCCCAGGTTGAG<br>CCCGGGGATTTCACATCTGACTTAACAAACCGCCTGCGTGCGCTTTACGCCCAGTAATTCCGATTAACGCTT<br>GCACCCTCCGTATTACCGCGGCTGCTGGCACGGAGTTAGCCGGTGCTTCTTCTGCGGGTAACGTCAATGAGC<br>AAAGGTATTAACTTTACTCCCTTCCTCCCCGCTGAAAGTACTTTACAACCCGAAGGCCTTCTTCATACACGC<br>GGCATGGCTGCATCAGGCTTGCGCCCATTGTGCAATATTCCCCACTGCTGCCTCCCGTAGGAGTCTGGACCG<br>TGTCTCAGTTCCAGTGTGGCTGGTCATCCTCTCAGACCAGCTAGGGATCGTCGCCTAGGTGAGCCGTTACCC<br>CACCTACTAGCTAATCCCATCTGGGCACATCCGATGGCAAGAGGCCCGAAGGTCCCCCTCTTTGGTCTTGCG<br>ACGTTATGCGGTATTAGCTACCGTTTCCAGTAGTTATCCCCCTCCATCAGGCAGTTTCCCAGACATTACTCA<br>CCCGTCCGCCACTCGTCAGCGAAACAGCAAGCTGTTTCCTGTTACCGTTCGACTGCATGTGTAGGCCTCCCC
Serratia liquefaciens	GU586145.1	GCCTAACACATGCAAGTCGAGCGGTAGCACAGGAAGCTTGCTCCTGGGTGACAAGCGGCGGACGGGTGAG<br>TAATGTCTGGGAAACTGCCTGATGGAGGGGGATAACTACTGGAAACGGTATCTAATACCGCATAACGTCTA<br>CGGACCAAAGTGGGGGACCTTCTGGCCTCATGCCATCACATGTGCCCAGATGGGATTATCTATTAGGTGGG<br>GTAATGGGTCACCTATGCGACAATCCCTATCTGGGCTGAGAGGATGACCACCCACACTGGAACTGAGACAC<br>GGTCCACACTCCTACGGGAGGCAGCAGTGGGGAATATTGCGCAATGGGCGCAAGCCTGATGCACCCATGCC<br>CCGTGTGTGAAAAAAGCCTTCTGGTTGTAAAGCACTTTCTCCGAGGAGGAAAGGGATTGTGTTAATATCAC<br>ATTGTATTGACGTTACTCTCACAAAAAACACCGGCTAACTCCGTGCCACCACCCGCGGTAATACAGAGGGT<br>GCGAGCGTTAATCTCAATTACTGGGCGTAAAGCGCACGCACGCGGTTTGTTAAGTCACATATGAAATCCCC<br>GCGCTTAACGTGGGAACTGCGTTTGAAACTGTGAAGCTATAGTCTTGTGTAGGGGGGTATAATTCTACGTGT<br>ATCGCTGAGATGCGTATAGATCTGGAGGAGTACCGGTGTCGAAAGCGGCCCCCTGTACAAAGACTGACACT<br>CACGTGCGAAAGCGTGTGGAGCACACACGATTAGATACCCTGGTAGTCCACGCTGTAAACGATGTCGACTT

	GGAGGTTGTGCCCTTGAGGCGTGGCTTCCGGAGCTAACGCGTTAAGTCGACCGCCTGGGGAGTACGGCCGC<br>AAGGTTAAAACTCAAATGAATTGACGGGGGCCCGCACAAGCGGTGGAGCATGTGGTTTAATTCGATGCAAC<br>GCGAAGAACCTTACCTACTCTTGACATCCAGAGAATTCGCTAGAGATAGCTTAGTGCCTTCGGGAACTCTG<br>AGACAGGTGCTGCATGGCTGTCGTCAGCTCGTGTTGTGAAATGTTGGGTTAAGTCCCGCAACGAGCGCAAC<br>CCTTATCCTTTGTTGCCAGCACGTARTGGTGGGAACTCAAAGGAGACTGCCGGTGATAAACCGGAGGAAGG<br>TGGGGATGACGTCAAGTCATCATGGCCCTTACGAGTAGGGCTACACACGTGCTACAATGGCGTATACAAAG<br>AGAAGCGAACTCGCGAGAGCAAGCGGACCTCATAAAGTACGTCGTAGTCCGGATCGGAGTCTGCAACTCG<br>ACTCCGTGAAGTCGGAATCGCTAGTAATCGTAGATCAGAATGCTACGGTGAATACGTTCCCGGGCCTTGTA<br>CACACCGCCCGTCACACCATGGGAGTGGGTTGCAAAAGAAGTAGGTAGCTTAACCTTCGGGAGGGCGCTTA<br>CCACT

RESEARCH ETHICS APPROVAL

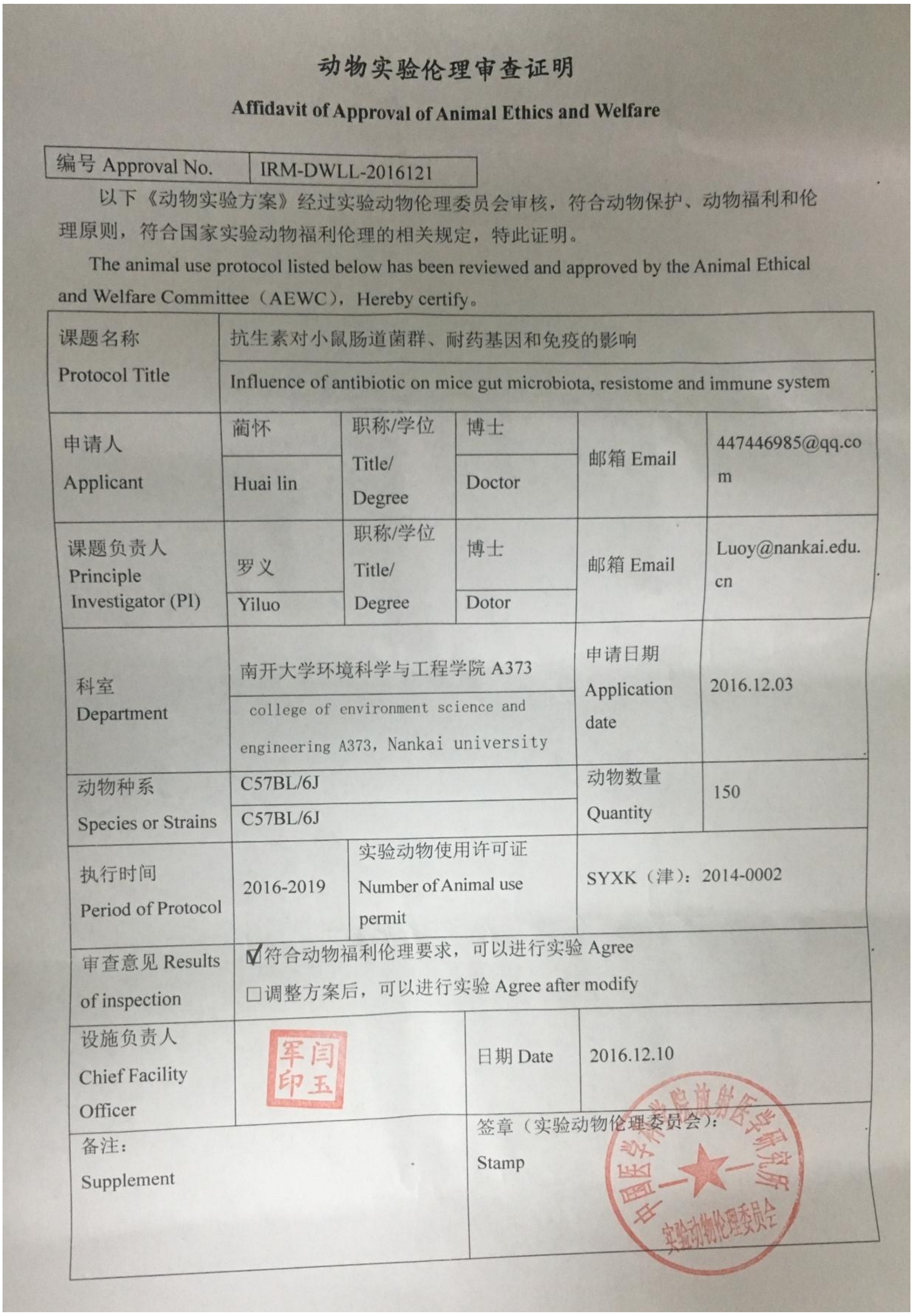

动物实验伦理审查证明

Affidavit of Approval of Animal Ethics and Welfare

编号 Approval No.	IRM-DWLL-2016121

以下《动物实验方案》经过实验动物伦理委员会审核，符合动物保护、动物福利和伦理原则，符合国家实验动物福利伦理的相关规定，特此证明。

The animal use protocol listed below has been reviewed and approved by the Animal Ethical and Welfare Committee（AEWC），Hereby certify。

课题名称 Protocol Title	抗生素对小鼠肠道菌群、耐药基因和免疫的影响<br>Influence of antibiotic on mice gut microbiota, resistome and immune system				
申请人 Applicant	蔺怀<br>Huai lin	职称/学位 Title/ Degree	博士<br>Doctor	邮箱 Email	447446985@qq.com
课题负责人 Principle Investigator (PI)	罗义<br>Yiluo	职称/学位 Title/ Degree	博士<br>Dotor	邮箱 Email	Luoy@nankai.edu.cn
科室 Department	南开大学环境科学与工程学院 A373<br>college of environment science and engineering A373，Nankai university			申请日期 Application date	2016.12.03
动物种系 Species or Strains	C57BL/6J<br>C57BL/6J			动物数量 Quantity	150
执行时间 Period of Protocol	2016-2019	实验动物使用许可证 Number of Animal use permit		SYXK（津）：2014-0002	
审查意见 Results of inspection	☑符合动物福利伦理要求，可以进行实验 Agree<br>☐调整方案后，可以进行实验 Agree after modify				
设施负责人 Chief Facility Officer	闫玉军印		日期 Date	2016.12.10	
备注：Supplement			签章（实验动物伦理委员会）：Stamp<br>中国医学科学院放射医学研究所 实验动物伦理委员会		
